# Ubiquitous genome streamlined *Acidobacteriota* in freshwater environments

**DOI:** 10.1093/ismeco/ycae124

**Published:** 2024-10-22

**Authors:** Hon Lun Wong, Paul-Adrian Bulzu, Rohit Ghai, Maria-Cecilia Chiriac, Michaela M Salcher

**Affiliations:** Department of Aquatic Microbial Ecology, Institute of Hydrobiology, Biology Centre of the Czech Academy of Sciences, Na Sadkach 7, 37005 České Budějovice, Czech Republic; Department of Biogeochemistry, Max Planck Institute for Marine Microbiology, Celsiusstraße 1, 28359 Bremen, Germany; Department of Aquatic Microbial Ecology, Institute of Hydrobiology, Biology Centre of the Czech Academy of Sciences, Na Sadkach 7, 37005 České Budějovice, Czech Republic; Department of Aquatic Microbial Ecology, Institute of Hydrobiology, Biology Centre of the Czech Academy of Sciences, Na Sadkach 7, 37005 České Budějovice, Czech Republic; Department of Aquatic Microbial Ecology, Institute of Hydrobiology, Biology Centre of the Czech Academy of Sciences, Na Sadkach 7, 37005 České Budějovice, Czech Republic; Department of Aquatic Microbial Ecology, Institute of Hydrobiology, Biology Centre of the Czech Academy of Sciences, Na Sadkach 7, 37005 České Budějovice, Czech Republic

**Keywords:** genome streamlined bacteria, aquatic microbial ecology, genome streamlined *Acidobacteriota* metagenomics, freshwater lakes, *Acidiparvus*

## Abstract

*Acidobacteriota* are abundant in soil, peatlands, and sediments, but their ecology in freshwater environments remains understudied. UBA12189, an *Acidobacteriota* genus, is an uncultivated, genome-streamlined lineage with a small genome size found in aquatic environments where detailed genomic analyses are lacking. Here, we analyzed 66 MAGs of UBA12189 (including one complete genome) from freshwater lakes and rivers in Europe, North America, and Asia. UBA12189 has small genome sizes (<1.4 Mbp), low GC content, and a highly diverse pangenome. In freshwater lakes, this bacterial lineage is abundant from the surface waters (epilimnion) down to a 300-m depth (hypolimnion). UBA12189 appears to be free-living from CARD-FISH analysis. When compared to other genome-streamlined bacteria such as *Nanopelagicales* and *Methylopumilus*, genome reduction has caused UBA12189 to have a more limited metabolic repertoire in carbon, sulfur, and nitrogen metabolisms, limited numbers of membrane transporters, as well as a higher degree of auxotrophy for various amino acids, vitamins, and reduced sulfur. Despite having reduced genomes, UBA12189 encodes proteorhodopsin, complete biosynthesis pathways for heme and vitamin K_2_, cbb_3_-type cytochrome *c* oxidases, and heme-requiring enzymes. These genes may give a selective advantage during the genome streamlining process. We propose the new genus *Acidiparvus*, with two new species named “*A. lacustris*” and “*A. fluvialis*”. *Acidiparvus* is the first described genome-streamlined lineage under the phylum *Acidobacteriota*, which is a free-living, slow-growing scavenger in freshwater environments.

## Introduction

Genome streamlined bacteria have reduced genomes caused by evolutionary selection as an adaptation to nutrient-limited environments, such as oceans and freshwater lakes [1]. These small genome-sized microorganisms have been found under different taxa, such as marine and freshwater *Pelagibacterales* (*Pelagibacter* and *Fonsibacter, Alphaproteobacteria*) [2, 3], OM43 and *Methylopumilus* (*Burkholderiales*) [4, 5], *Nanopelagicales*, *Actinomarinales*, and *Rhodoluna* (*Actinobacteriota*) [6–10].

*Acidobacteriota* is a bacterial phylum primarily studied in soils and peatlands [11]. These microbes usually have large genome sizes (4.9–6.7 Mb) and versatile metabolic capacities, allowing them to break down complex biopolymers and participate in sulfur, carbon, and iron cycling [11–14]. *Acidobacteriota* are also common in freshwater lakes, especially in acidic systems and sediments [15–17]. However, there are limited studies on *Acidobacteriota* in circumneutral freshwater habitats that are not associated with sediments. A recent study of freshwater *Acidobacteriota* assembled from the water column revealed lineages co-occurring with *Cyanobacteria* and being heterotrophs that can utilize a broad range of exopolysaccharides [18].

The *Acidobacteriota* family *Holophagaceae* contains the described genera *Holophaga* [19] and *Geothrix* [20], and several placeholder names have been established for uncultivated genera (JACQZU01, JADJVL01, JAFDVN01, WRHW01, and UBA12189) [21]. UBA12189 is the only *Acidobacteriota* lineage with an ultra-small genome size (estimated genome size <1.4 Mb). So far, a total of 51 metagenome-assembled genomes (MAGs), including 44 high-quality MAGs (>70% completeness, <5% contamination) have been found in various nonsediment aquatic environments such as the Laurentian Great Lakes [22], Lake Biwa [23, 24], an alpine monomictic lake (Lake Fuxian) [25], Lake Erken [26], and an estuary at Columbia River [27]. However, no detailed genomic studies were done on this bacterial lineage.

Here, we report an additional 22 high-quality MAGs obtained from 8 European freshwater lakes, showing that this genus is fundamentally different from other genera under the same family *Holophagaceae*. UBA12189 shares common traits of aquatic, free-living genome streamlined bacteria such as low GC content, high coding density, low number of two-component systems, and presence of rhodopsins. Whilst other freshwater genome-streamlined lineages (i.e. *Fonsibacter*, *Methylopumilus*, *Nanopelagicales*) appear to compensate lacking biosynthesis pathways for amino acids and vitamins with transporters [3, 5, 10], the number of membrane transporters is lower in UBA12189 and numerous metabolic pathways are absent. Despite having extremely reduced genomes, UBA12189 encodes proteorhodopsins and complete biosynthesis pathways for heme and vitamin K_2_. The presence of cbb_3_-type cytochrome *c* oxidase suggests this bacterial lineage can survive in both microaerobic and anaerobic environments [28]. This is the first report of a genome streamlined lineage under the phylum *Acidobacteriota* that we tentatively name *Acidiparvus*, which is a free-living auxotroph with extremely small genome size and very limited metabolic capacity.

## Materials and methods

### Sample collection, DNA extraction, and sequencing

Freshwater samples were collected from eight lakes in Europe (Supplementary Table S1). Sampling details were described in previous studies [29, 30]. For each lake, approximately 20 l of water samples were collected from the epilimnion and hypolimnion. Subsamples (100 ml) were immediately fixed with formaldehyde (2% final concentration) for flow cytometric counting and the preparation of fluorescence *in situ* hybridization followed by catalyzed reporter deposition (CARD-FISH) filters as detailed in Chiriac *et al*. (2022) [30]. The remaining water was sequentially filtered through a 20-μm mesh plankton net to remove larger organisms, followed by 5- and 0.22-μm polyethersulfone membrane filters (Millipore, Merck, Darmstadt, DE) until completely clogged. These filters were immediately immersed in DNA/RNA shield (Zymo Research, Irvine, CA, USA) and stored at −80°C. The 0.22 μm filters were then cut into small pieces with sterile scissors, followed by DNA purification and extraction using the ZR Soil Microbe DNA MiniPrepTM kit (Zymo Research, Irvine, CA, USA) according to the manufacturer’s instructions. DNA samples were then sent for shotgun metagenomic sequencing (PE150) utilizing the Illumina NovaSeq 6000 and NextSeq 500 platform. Additionally, several samples were sequenced on a Nanopore PromethION platform using the SQK-LSK110 ligation sequencing kit (Oxford Nanopore, Oxford, UK) with R9.4.1 flow cells.

### Data quality control, metagenomic assembly, long-read sequencing assembly, and binning

The pipeline of short-read sequences was described in detail by Chiriac *et al*. (2022) [30]. To remove poor-quality reads from the raw data, bbduk.sh script (qtrim = rl, trimq = 18) was employed [31]. phiX, p-Fosil2 control reads and Illumina adaptors were also removed. Subsequently, the processed reads were assembled de novo using MEGAHIT v1.1.4.2 with the selection of k-mers: 29, 49, 69, 89, 109, 119, 129, and 149 [32]. Assembled contigs of <3 kb were removed before binning. The resulting contigs were mapped to the quality reads, then binning was performed using MetaBAT v2.15 [33]. To minimize contamination, gene prediction was performed with Prodigal v2.6.3 [34] and the taxonomy of genes was determined by aligning each gene against GTDB r89 and UniProt release 2020-02 with MMseq2 (e-value <1e^−3^, similarity >10%, coverage >10% bitscore >50), retrieving the taxonomy of the best hit gene. Contigs with <30% of the genes assigned to the dominant taxonomy for their bin were considered contaminants and were discarded [35]. VirSorter [36] and VIBRANT [37] were used to predict viral sequences, in which contigs having >25% of viral genes were eliminated. The MAGs were dereplicated using dRep at average nucleotide identity (ANI) >99% [38]. Taxonomy classification was performed using GTDB-tk v2.3.2 [21] against GTDB database version r207 and CheckM2 [39] was employed to estimate the completeness and contamination of the MAGs. MAGs under the genus UBA12189 (family *Holophagaceae)* with completeness >70% and contamination <5% were selected for further analyses.

For long-read sequences, after quality control and trimming as described above, the reads were used to generate a Burrows–Wheeler Transform (BWT) required for polishing noisy long-reads, according to the ropebwt2 construction approach [40]. Nanopore base called long-reads with a Q score ≥ 8 was subjected to adapter and barcode trimming by Porechop [41]. These reads were further polished using the generated Illumina BWT with FMLRC2 v0.1.8 with default parameters [42]. Polished long-reads were assembled using Flye v2.9.1-b1780 (−-nano-corr–meta–no-alt-contigs) [43] and contigs >10 kb were binned to MAGs as described above. This resulted in two long-read sequencing MAGs classified as UBA12189 (ZE-13nov19-LR-8 and ZE-03apr19-LR-56).

### Genome annotation

Proteins were predicted using Prodigal v2.6.3 while rna_hmm3 [44] and tRNAscan-SE [45] were used to predict rRNA and tRNA coding sequences, respectively. Subsequently, protein annotations were performed utilizing an in-house pipeline that involved the application of hmmsearch [46] against collections of COG [47], TIGRFAM [48], Pfam [49], hidden Markov models (HMMs), and the KOALA algorithm against a non-redundant KEGG database (KOfam release 1.3.0 [50]). Gene prediction was only included if the protein had a minimum coverage of 65% for its entire length in the HMM model, with an e-value threshold of 1e^−3^. Proteins were also annotated using InterProScan [51] with default parameters. Furthermore, carbohydrate-active enzymes (CAZy) annotation was performed using hmmscan [46] and the dbCAN CAZyme domain HMM database v10 (release date 17 August 2021) [52]. A figure of metabolic pathways was created with biorender.com with publication license YM2759VU7C.

### Fragment recruitment and doubling time estimation

A total of 58 metagenomes from nine lakes and 35 metagenomes from rivers were utilized for fragment recruitment (Supplementary Tables S1 and S2). Barrnap (http://www.vicbioinformatics.com/software.barrnap.shtml) was used to screen for rRNA sequences in UBA12189 MAGs, which were then masked to avoid biases. For each metagenome, 20 million quality-filtered reads were mapped against the MAGs using MMseqs2 (minimum alignment identity = 95%, minimum alignment length = 50%, minimum coverage of the read = 90%) [53]. The resulting number of hits was employed to compute coverage per Gb values, which enabled the determination of normalized abundances that could be compared across different MAGs and metagenomes, where only 53 metagenomes having an average of >0.1 coverage per Gb were included (Supplementary Table S2). The rate of bacterial replication was estimated using the GRiD multiplex module with default options (Supplementary Table S3) [54].

### Phylogenomic analysis

Apart from the 22 MAGs assembled in the current study, 44 high-quality MAGs (>70% completeness, <5% contamination) from public databases (GTDB r207 and JGI), 237 other MAGs under the family *Holophagaceae*, which are species representatives, were also obtained from GTDB r207 [21], JGI IMG (Joint Genome Institute – Integrated Microbial Genomes) [55] and a comprehensive metagenomics dataset from freshwater environments [26]. Three *Hydrogenedentota* MAGs were used as outgroups. A set of 46 single-copy genes (SCGs; the minimum number of common SCGs in every MAG) were selected to construct the phylogenetic tree (Supplementary Table S4). Each SCG amino acid sequence was aligned using PRANK [56]. The resulting alignments were then trimmed with BMGE with the parameters “-m BLOSUM62 -t AA -g 0.5 -b 5” and concatenated [57]. A maximum-likelihood tree was constructed using IQ-TREE2 [58] with 1000 bootstraps replication, with the model LG + I + G suggested by the ModelFinder [59], then visualized with iTOL [60].

To determine the genetic similarity between the UBA12189 MAGs, ANI, and average amino acid identity (AAI) were calculated using established thresholds [61, 62]. Additionally, the percentage of conserved proteins was determined through all-vs-all comparisons, with a requirement of over 50% identity and coverage (as described in Qin *et al*., 2014; [63]). The core- and pan-genomes were computed by comparing all proteins within each genome using BLASTP. An orthologue was defined as having at least 50% identity and 50% coverage. The description of a novel genus and two species, following SeqCode nomenclature, is available in Supplementary Text [64].

### Comparative genomic analysis

The aforementioned 237 *Holophagaceae* MAGs were dereplicated at 95% using dRep [38], resulting in 41 representative MAGs and culture genomes for comparative analysis. Thirty-three genome-streamlined *Nanopelagicales* [10] and *Methylopumilus* [65] were also selected for comparative genomic analysis (Supplementary Table S5). Selection criteria and genome annotation process are elaborated in the Supplementary Text.

### Phylogenetic analysis of rhodopsins

All UBA12189 MAGs were scanned for rhodopsins. We first predicted proteins using Prodigal v2.6.3 [34] and then searched for significant hits to rhodopsin HMMs, specifically PFAM HMMs corresponding to bac_rhodopsin and heliorhodopsin, employing hmmsearch [46]. All putative hits with sequences shorter than 150 amino acids and *P*-values > 1e^−2^ were discarded. Protein sequences matching the aforementioned criteria were selected and compared to a rhodopsin database, including all known rhodopsin sequences in UniProt and GTDB using MMseqs [35]. For each query sequence, we selected the top 50 hits, which were then aligned with MAFFT (−-localpair–maxiterate 1000) [66]. Polyphobius [67] was employed to predict the number of transmembrane helices in the resulting multiple sequence alignments (seven for canonical rhodopsins), orientation of the rhodopsin (i.e. Type I rhodopsins, heliorhodopsins), and the motifs for retinal binding site in transmembrane helix 7 (DxxxK for Type I rhodopsins vs. SxxxK for heliorhodopsins). The resulting rhodopsin sequences were aligned with 2199 representative rhodopsin sequences from a previous study [68] with PASTA [69]. A phylogenetic tree was constructed with IQ-TREE2 (-B 1000, −-alrt 1000) [58], with ultrafast bootstrapping and the LG + I + G4 model suggested as the best-fit model by ModelFinder [59]. Rhodopsin contigs were visualized with gene graphics [70].

BLASTP of the genomes, neighboring genes of rhodopsins, and uroporphyrinogen-III synthase (*hemD*) were done with the NCBI RefSeq non-redundant (nr) protein database release 218. *Acidobacteriota hemD* genes from NCBI were used as reference sequences. Sequences were aligned with MAFFT [66] and the phylogenetic tree was constructed with IQ-TREE2 [58] with 1000 bootstrap replications and evolutionary model LG + F + G4.

### Probe design and fluorescence *in situ* hybridization followed by catalyzed reporter deposition

An oligonucleotide probe targeting the 16S rRNA of UBA12189 was designed as previously described [30, 71]. 16S rRNA gene sequences of UBA12189 MAGs were aligned with the SINA web aligner [72] and imported to ARB [73] using SILVA SSU database RefNR99 release 138.1 [74]. A Randomized Axelerated Maximum Likelihood tree (RAxML, 100 bootstraps, GTR-GAMMA model) [75] was constructed including all near full-length sequences (>1200 bp) of the lineage “marine group Holophagales_Holophagaceae” that corresponds to UBA12189 and closely related reference sequences (Supplementary Fig. S1). Alignments were manually curated prior to tree construction. Probe design was done with the ARB tools Probe_Design and Probe_Match; candidate probes were further evaluated in silico with the online tool MathFish [76]. Formamide concentrations for probe acido826 (AACGCCCACTACACCAAG) were tested on CARD-FISH filters from Lake Zurich (5% stepwise increase between 45 and 65% formamide) until stringent conditions were achieved at 55% formamide (i.e. stable number of hybridized cells at highest possible signal intensities).

CARD-FISH was carried out for lakes with high recruitment values (i.e. Lake Zurich, Lake Maggiore, and Lake Biwa). Samples were collected from multiple depth layers representing the epilimnion (0–20 m, four to eight samples) and hypolimnion (30 m to maximum depth, tow to five samples) during Spring and Autumn 2019 (Lake Zurich and Lake Maggiore) and in Autumn 2022 (Lake Biwa). CARD-FISH was done and evaluated by automated microscopy (Zeiss Imager.Z2 equipped with a Colibri LED light system and filter sets for 4′,6-diamidino-2-phenylindole (DAPI), fluorescein, and autofluorescence; Carl Zeiss, Oberkochen, DE) as previously described [5, 30]. Total prokaryotic abundances were determined by flow cytometry using a CytoFlex instrument (Beckman Coulter, Brea, CA, USA) after staining with Sybr Green (0.5 × final concentration) and percentages obtained by CARD-FISH were used to calculate total abundances of UBA12189.

## Results and discussion

### Phylogenomic characterization, diversity, and distribution of UBA12189

**Figure 1:**
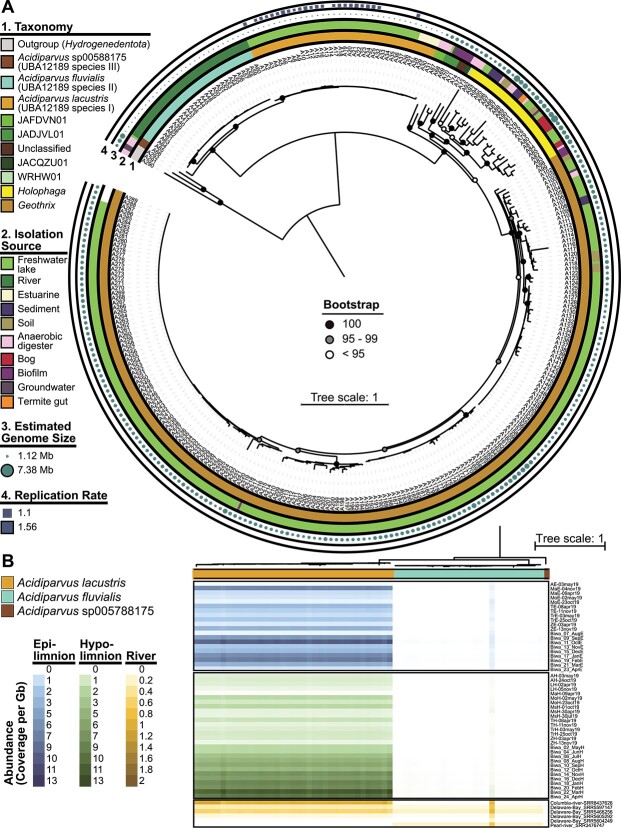
Phylogenetic tree of *Holophagaceae*. Maximum likelihood phylogenetic tree with LG + I + G model under the *Holophagaceae* family of all GTDB species representatives, based on 46 concatenated SCGs (Supplementary Table S4). Hydrogenedentota was used as the outgroup. (A) Phylogenetic tree with an outer panel showing taxonomy, source of metagenome, estimated genome size, and estimated replication rate using GRiD. Labels with corresponding MAG names are listed in Supplementary Table S4. GRiD values were only estimated for the MAGs from our samples. (B) Relative abundance of the MAGs in metagenomics read recruitment expressed as coverage per Gb of the metagenome.

A total of 66 MAGs (63 MAGs from short-read sequencing and three MAGs from long-read sequencing) of the genus UBA12189 were investigated in this study. This genus, alongside *Holophaga*, *Geothrix* and four novel genera (JADJVL01, JAFDVN01, JACQZU01, and WRHW01) comprise the family *Holophagaceae*. All MAGs have >70% completeness and <5% contamination, including one complete, circular genome (sbin449) (Supplementary Table S5). Most of the MAGs were obtained from freshwater environments, except for four MAGs assembled from the Columbia River estuary. All MAGs have common features of free-living genome streamlined lineages, such as small, estimated genome sizes ranging from 1.12 to 1.37 Mb, high coding density ranging from 92.2% to 95.3% and low GC contents ranging from 44.3% to 48.7% comparing to other *Acidobacteriota* (Supplementary Tables S5 and S6) [77]. These are the smallest recorded genome sizes of *Acidobacteriota* so far and this lineage is the only genus with streamlined genomes under the family *Holophagaceae* (Supplementary Text). A phylogenetic tree of all available *Holophagaceae* genomes resulted in three separated clades of UBA12189 (Fig. 1, Supplementary Table S5). Interestingly, MAGs from the current study are in the same phylogenetic clade (species I) together with MAGs assembled from Lake Biwa and estuarine environments (Fig. 1), while species II contains MAGs exclusively from rivers and species III consists of one MAG assembled from alpine Lake Fuxian. ANI, and AAI also suggest the presence of three different species (ANI < 95%) within the same genus (AAI > 65%) [62] (Supplementary Figs S2 and S3). We therefore propose that UBA12189 is a novel genus consisting of three species that we tentatively name *Acidiparvus*. Species I and II are designated *Acidiparvus lacustris* and *Acidiparvus fluvialis,* because of their occurrence in freshwater lakes and rivers, respectively. Species III did not meet the criteria to be named as species; we thereby refer to it as *Acidiparuvs sp005788175* based on current GTDB classification (Supplementary Text).

To estimate the abundance of *Acidiparvus* in freshwater metagenomes, metagenomic fragment recruitment was employed (Fig. 1). Coverage per Gb ranges from 0 to 13 (Supplementary Table S2), with the highest coverage obtained from the hypolimnion of Lake Biwa during mixis in Spring (March–April) and the epilimnion in Autumn and Winter (October and February). On average, *A. lacustris* is most abundant in Lake Biwa (Japan), followed by Lake Maggiore (Italy) and Lake Zurich (Switzerland). The results suggest that *A. lacustris* is a flexible species that can inhabit both the surface waters and hypolimnion water columns of large lakes across distant geographical locations. Furthermore, results show that only *A. lacustris* is abundant in the lakes sampled in the current study, while *A. fluvialis* and *A. sp 005788175* were practically absent. Although all *A. fluvialis* MAGs were assembled from metagenomes sampled from rivers, this species was only occasionally present in lotic environments, and the preferred habitat of *A. sp005788175* is currently unknown (Fig. 1). Compared to the abundance of other genome-streamlined microbes, *Acidiparvus* has a similar abundance as candidate phyla radiation (CPR; 0–14.4 coverage per Gb) [30], while lower than *Methylopumilus* (4.9–48 in Lake Zurich, 2.1–19.1 in Lake Biwa) [5] and *Nanopelagicales* (6.1–116 in Lake Zurich) [10]. Genome replication rates in lake-dwelling *Acidiparvus* were estimated with GRiD based on ori/ter values, ranging from 1.1 to 1.56, indicating slow growth and replication rates at the sampling timepoints (Fig. 1, Supplementary Table S3).

### *Acidiparvus* appears to be free-living

**Figure 2:**
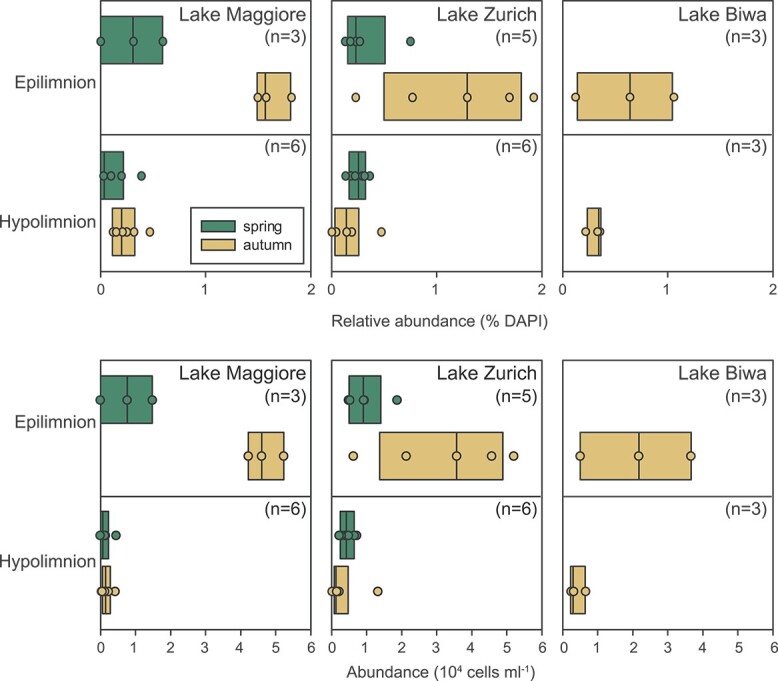
Seasonal abundance of UBA12189. Abundance of *Acidiparvus* in different seasons determined by CARD-FISH and flow cytometry. (A–C) Relative average abundances (% of all DAPI stained prokaryotes) of *Acidiparvus* in the epi- and hypolimnion of Lakes Maggiore, Zurich, and Biwa during spring and autumn, respectively. (D–F) Total average abundances (10^4^ cells ml^−1^) of *Acidiparvus* in the epi- and hypolimnion of Lake Maggiore, Zurich, and Biwa during spring and autumn, respectively. Abundances were calculated by multiplying relative abundances with total prokaryotic counts determined by flow cytometry. Boxplots display the median (central line) and ranges (boxes) of multiple depth layers representing the epilimnion (0–10 m) and hypolimnion (13 m to maximum depth); the number of samples for each plot is given in brackets. Dots represent all individual samples. A full figure containing all depth discrete samples is available as Supplementary Fig. S4.

**Figure 3:**
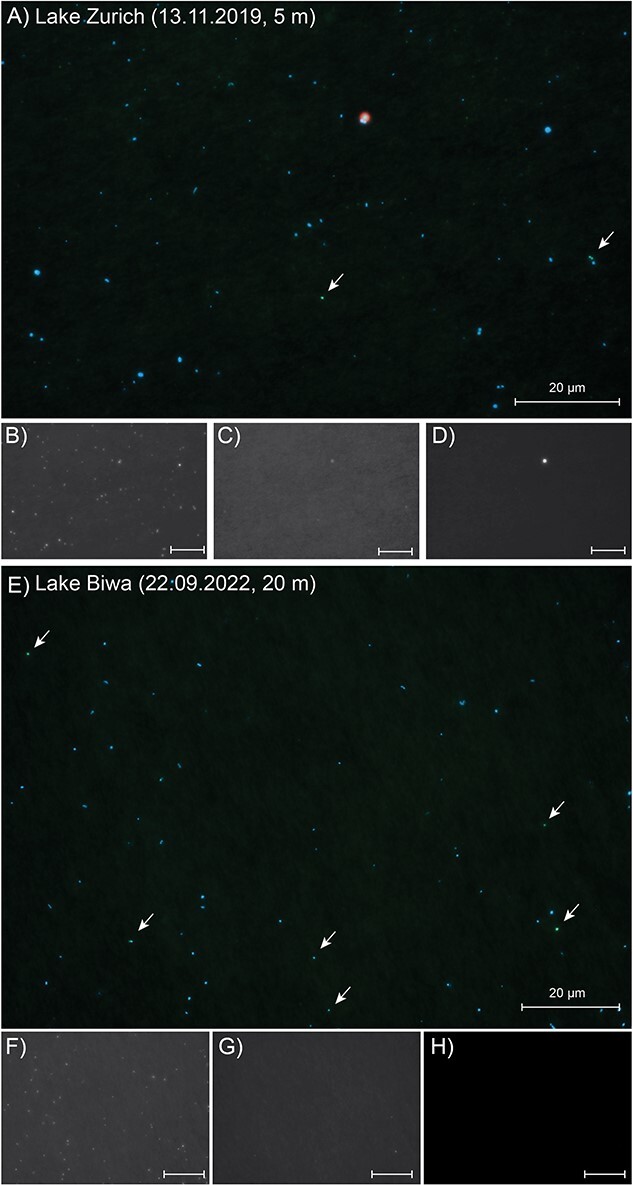
CARD-FISH imaging of *Acidiparvus* in Lake Zurich and Biwa. (A, E) Overlay images of CARD-FISH (green), DAPI (blue), and autofluorescence (red) signals from samples from Lake Zurich (A) 5 m depth, 13 November 2019) and Lake Biwa (E) 22 September 2022). (B–D, F–H) Individual images of DAPI-stained prokaryotes (B, F), CARD-FISH stained *Acidipde noviarvus* (C, G), and autofluorescence (D, H) from the same field of view. *Acidiparvus* cells are highlighted by arrows. The scale bar in all images represents 20 μm.

CARD-FISH with probe acido826 show that *Acidiparvus* can account for up to 1.9% of all prokaryotes with abundances of up to 5.2 × 10^4^ cells ml^−1^ in lakes Zurich, Maggiore, and Biwa (Fig. 2, Supplementary Fig. S4). *Acidiparvus* have maximum abundances in the epilimnion and gradually decreases with depth with higher densities during autumn compared to spring. CARD-FISH microscopy images show that all *Acidiparvus* are very small and free-living; they were never found attached or in close vicinity to other microorganisms (Fig. 3, Supplementary Fig. S5). To further examine if they have potential associations with other microorganisms, we performed BLASTP to identify any traces of horizontal gene transfer (HGT). All but one MAG (UppL3-bin-119) have >90% of the genome of *Acidobacteriota* origin (to be specific, UBA12189), suggesting no evidence of potential recent HGT events (Supplementary Table S7). Furthermore, without a complete type II secretion system, and the complete absence of genes involved in type III, type IV, and type VI secretion systems, there is no concrete evidence of any potential host association (Supplementary Text). This reinforces the free-living aspect of *Acidiparvus*. However, we found extremely low metabolic versatility in the MAGs suggesting this bacterium cannot live alone, which will be described in detail in the following paragraphs.

### The genomic repertoire of *Acidiparvus*

**Figure 4:**
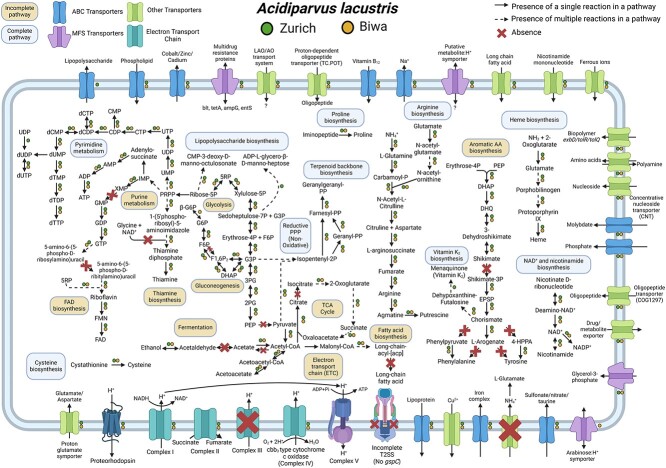
Metabolic map reconstruction of two long-read *A. lacustris* genomes assembled from Lake Zurich (ZE-13nov19-LR-8) and Lake Biwa (sbin449), the latter one being a complete genome. Abbreviations: ABC, ATP-binding cassette; MFS, major facilitator superfamily; 1,3BPG, 1,3-bisphosphoglycerate; 2PG, 2-phosphoglycerate; 3PG, 3-phosphoglycerate; 5RP, ribulose 5-phosphate; 6PG, 6-phosphogluconate; 6PGL, 6-phoshogluconolactone; DAHP, 2-dehydro-3-deoxy-D-arabino-heptonate 7-phosphate; DHAP, dihydroxyacetone phosphate; DHQ, 3-dehydroquinate; dTDP, deoxythymidine diphosphate; dTDP-DXH, dTDP-6-deoxy-D-xylo-4-hexulose; EPSP, 5-enolpyruvylshikimate-3-phosphate; F1,6P2, fructose 1,6-bisphosphate; F6P, fructose 6-phosphate; FAD, flavin adenine dinucleotide; FMN, flavin mononucleotide; G3P, glyceraldehyde 3-phosphate; G6P, glucose 6-phosphate; HTPA, (2S,4S)-4-hydroxy-2,3,4,5-tetrahydrodipicolinic acid; IMP, inosine monophosphate; LPS, lipopolysaccharides; NAD^+^, nicotinamide adenine dinucleotide; P, phosphate; PEP, phosphoenolpyruvate; PPi, pyrophosphate; PPP, pentose phosphate pathway; PPRP, phosphoribosyl pyrophosphate; TDO, dTDP-4-oxo-L-rhamnose; THF, tetrahydrofolate; TXN, thioredoxin; TXN-S-S, thioredoxin disulfide; XMP, xanthosine monophosphate; ETC, electron transport chain; TCA, tricarboxylic acid.

**Figure 5:**
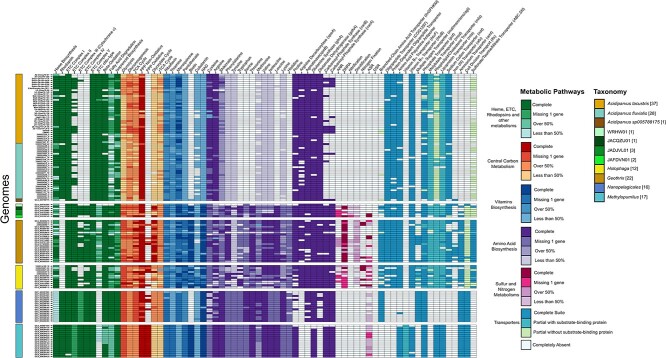
Presence and absence of metabolic pathways. Heatmap showing the presence and absence of certain metabolic pathways in *Acidiparvus* compared to other lineages of *Holophagaceae* and genome streamlined lineages such as *Nanopelagicales* and *Methylopumilus*. Left panels indicate different metabolic pathways, while color panel on the right represents taxonomy of MAGs. Bracketed numbers indicate the number of genomes analyzed in this study. Abbreviations: ETC, electron transport chain; TCA, tricarboxylate acid; PPP, pentose phosphate pathway; NAD, nicotinamide adenine dinucleotide; ANR, assimilatory nitrate reduction; DNRA, dissimilatory nitrate reduction; ASR, assimilatory sulfate reduction; DSR, dissimilatory sulfate reduction.

We conducted comparative genomic analyses of 66 *Acidiparvus* genomes, including one circular, complete genome (sbin449), to obtain a comprehensive view of the genomic repertoire and metabolic potential of this *Acidobacteriota* lineage (Figs 4 and 5). Since *Acidiparvus* have extremely limited metabolic capacity, we compared their genomes to similar aquatic genome streamlined microbes such as *Nanopelagicales* [10] and *Methylopumilus* [65] as well as to other *Acidobacteriota* in this study.

*Acidiparvus* contains genes for glycolysis, gluconeogenesis, and the tricarboxylic acid (TCA) cycle in various degrees of completeness and a full gene set for the non-oxidative phase of the pentose phosphate pathway (PPP) (Fig. 5, Supplementary Table S8). All the MAGs lack pyruvate kinase, rendering an incomplete glycolysis pathway in this bacterial lineage. As 6-phosphofructokinase is missing in all MAGs, alpha-D-glucose 6-phosphate could be converted to D-xylulose-5-phosphate followed by glyceraldehyde 3-phosphate (G3P) in the PPP, then fed back into the core glycolysis pathway (Fig. 4). Results showed that the MAGs encode genetic components involved in the non-oxidative phase of the PPP and glycolysis via the Embden-Meyerhof-Parnas pathway instead of the Entner-Doudoroff pathway (Figs 4 and 5). Unlike some members from the family *Holophagaceae*, all *Acidiparvus* MAGs lack glucose-6-phosphate dehydrogenase and 6-phosphogluconate dehydrogenase, rendering an incomplete oxidative phase of PPP in this lineage (Supplementary Text). Half of the MAGs encode genes for glycogen synthesis, suggesting the capacity to use glycogen as carbon and energy storage. In terms of CAZy, all MAGs encode glycosyltransferase family 2 and 4 (GT2 and GT4), indicating the potential ability for polysaccharide conversion and rhamnose degradation (Supplementary Table S9). Unlike other *Holophagaceae* members, none of the *Acidiparvus* encode a complete TCA cycle (Fig. 5). Furthermore, genes involved in fermentation, such as acetaldehyde dehydrogenase (*adhE*) and lactate dehydrogenase (LDH), were not identified in all genomes, while 64 MAGs encode alcohol dehydrogenase, suggesting a limited fermentative lifestyle.

None of the MAGs encode genes for assimilatory or dissimilatory sulfate or nitrate reduction, indicating a dependency on exogenous sources of reduced sulfur and nitrogen for cell metabolism and growth, which is also the case in other streamlined bacteria [10, 30, 78]. In contrast, most other *Holophagaceae* (JACZQ01, JAFLDN01, JANVDN01, *Geothrix, Holophaga*) have the ability to reduce and assimilate sulfate and nitrate and eight of them can fix nitrogen (Fig. 5 and Supplementary Text).

### Limited capacity for amino acid/vitamin biosynthesis and membrane transporters

*Acidiparvus* have highly restricted capacities for amino acid biosynthesis, which are limited to cysteine, arginine, and proline (Figs 4 and 5). While other *Holophagaceae* and other genome streamlined freshwater taxa have most pathways for amino acid synthesis (Fig. 5, Supplementary Text), *Acidiparvus* is auxotrophic for the majority of amino acids. Sixty-one MAGs contain genes for proline synthesis, while 50 MAGs are able to produce cysteine. Sixty-one MAGs have the complete pathway for arginine synthesis, while other members of *Holophagaceae, Nanopelagicales*, and *Methylopumulis* are lack N-acetylglutamate synthase to convert glutamate to N-acetylglutamate (Supplementary Tables S8 and S10). Compared to *Nanopelagicales* and *Methylopumilus*, *Acidiparvus* is the only genome streamlined genus to encode N-acetylornithine carbamoyltransferase (*argF*) and acetylornithine deacetylase (*argE*), allowing them to synthesize arginine from carbamoyl-P.

Cysteine is synthesized by cystathionine gamma-lyase instead of cysteine synthase; thus, no hydrogen sulfide is incorporated into the cells. Since no evidence of genes responsible for sulfate reduction or assimilation was identified, the mode of acquisition of reduced sulfur is unknown. As a notable difference from streamlined *Nanopelagicales* and *Methylopumilus*, *Acidiparvus* may take taurine and sulfonate as an external sulfur source since half of the MAGs encode sulfonate/Nitrate/Taurine transporters (*ABC.SN*) (Fig. 5). However, taurine/sulfonate degradation genes were not identified.

Most of the MAGs share transporters for phosphate, molybdate, sodium, phospholipid, and cobalamin, while *afu* iron (III) transporters are exclusively found in *A. sp005788175* (Fig. 5, Supplementary Table S8). Compared to *Nanopelagicales* and *Methylopumilus*, *Acidiparvus* is the only genome-streamlined lineage to encode sodium and molybdate transporter systems (*natAB*, *modABC*) (Fig. 5). Most MAGs encode the complete set of high-affinity *pstABCS* transport systems for phosphorus acquisition, (Fig. 5, Supplementary Table S8), suggesting that this lineage is well adapted since phosphorus is the primarily limiting nutrient in freshwater lakes [79, 80]. All *Acidiparvus* MAGs encode cobalamin (vitamin B_12_) transporter (*btuB*), with 51 MAGs having 2 copies of the gene.

Given that all *Acidiparvus* lack the ability to produce most amino acids, they must rely on external sources. However, amino acid transporters from the ABC family (*livGFHMK*, *oppABCDF*, *dppABCDF*) are absent in all MAGs. In comparison, other genome-streamlined amino acid auxotrophs such as *Nanopelagicales* contain branched amino-acid transporters (*livGFHMK*) (Fig. 5) [65]. Despite *Acidiparvus* lacking amino acid transporters, they encode homologs of oligopeptide transporters of the OPT superfamily (COG1297) and proton-dependent oligopeptide transporters of the POT superfamily (TC.POT; K03305) (Fig. 5). These putative oligopeptide transporters are also identified in all other *Holophagaceae* lineages but not in *Nanopelagicales* and *Methylopumilus*. Different from other prokaryotic OPTs, which usually have 16 transmembrane domains, most of the OPT homologs in *Acidiparvus* harbor 17 transmembrane domains (Supplementary Table S11). It was suggested that this transporter can act as both peptide and iron-siderophore transporters [81]. Most of the *Acidiparvus* MAGs also encode multiple copies of TC.APA basic amino acid/polyamine antiporters (K03294), allowing them to export polyamines in exchange for amino acids. Indeed, all *Acidiparvus* MAGs have at least one set of genes for polyamine production (*speAB*, agmatine, and putrescine production), potentially enabling them to produce and exchange polyamine for amino acids (Fig. 5). As for nitrogen uptake, unlike other *Holophagaceae* and genome streamlined lineages, urea transporter and cyanophycinase are absent in all *Acidiparvus* genomes, indicating that these are not the major nitrogen sources. Most of the MAGs encode glutamate dehydrogenase (*gdhA*), which allows them to convert ammonium to glutamate as a source for nitrogen assimilation. However, ammonium transporters (*amt*) were not identified in any of the *Acidiparvus* genomes, suggesting the presence of putative novel, currently unannotated proteins to import ammonium across the cell membrane.

Apart from being auxotroph for most of the amino acids, *Acidiparvus* are also auxotrophs for vitamins B_1_ (thiamin), B_3_ (nicotinamide adenine dinucleotide), B_6_ (pyridoxine), B_12_ (cobalamin), and B_7_ (biotin). However, *Acidiparvus* have complete biosynthesis pathway for vitamin K_2_ (menaquinone) and near-complete pathway for vitamin B_2_ (riboflavin) biosynthesis (Fig. 5). Not only does *Acidiparvus* have limited metabolic capacities and high degree of auxotrophy as described above, this *Acidobacteriota* lineage also lacks hydrogenases, cyanophycinases, CRISPR-Cas systems and biosynthetic gene clusters. With a very limited genome repertoire, as well as the small number of cell membrane transporters, this all points to a scenario where free-living *Acidiparvus* have a constrained cell metabolism that is highly specialized [6].

### Unique set of genes in *Acidiparvus* as adaptation to the sunlit epilimnion of lakes

Since *Acidiparvus* has low metabolic versatility and appears to lack several general molecular machineries for survival, we compared the genomes with other genera within the family *Holophagaceae* (*Geothrix*, *Holophaga*, JAFDVN01, JADJVL01, JACQZU01, and WRHW01) and other genome streamlined freshwater microbes to identify unique features of this genome streamlined genus.

**Figure 6:**
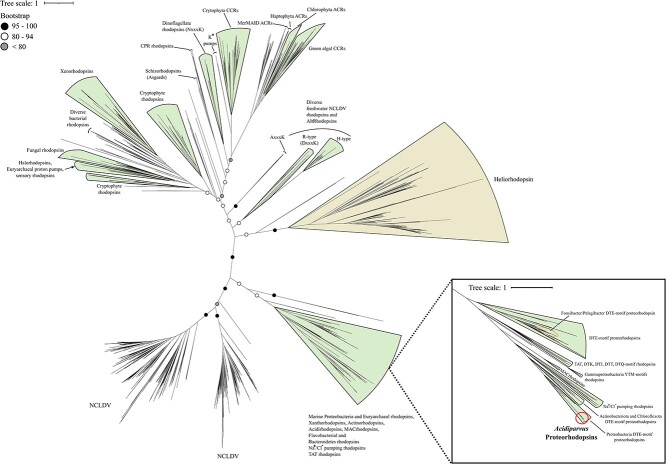
Phylogenetic tree of *Acidiparvus* proteorhodopsins. Maximum likelihood tree (LG + I + G4) for rhodopsin phylogeny. A total of 2261 sequences were used to generate the tree, including 62 rhodopsins identified in the current study and 2199 proteins from known rhodopsin families [67]. Red circle indicates proteorhodopsins identified in *Acidiparvus* MAGs in the current study. Abbreviations: ACR, anion channelrhodopsin; CCR, Cation channelrhodopsin; MerMAIDs, phylogenetically distinct anion-conducting channelrhodopsins; NCLDV, nucleocytoplasmic large DNA viruses; CPR, candidate phyla radiation.

Microbial rhodopsins are transmembrane proteins that are light-driven proton pumps for specific biological functions such as ATP generation, which is an adaptational feature in genome-streamlined bacteria found in the pelagial of oceans and lakes [77, 82]. *Acidiparvus* is the only genus in the family *Holophagaceae* to encode rhodopsins. A total of 62 rhodopsins were identified in the 66 MAGs. All the rhodopsins were predicted to be proteorhodopsins harboring DTE-motifs in helix C, which appear to form their own phylogenetic cluster branching off proteorhodopsins from other prokaryotic taxa, while phylogenetically closest to Alpha- and Gammaproteobacterial DTE-motif proteorhodopsins (Fig. 6). All proteorhodopsins contain leucine at amino acid position 105 and retinal-binding lysine in helix G, indicating they are green-light absorbing and light sensitive (Supplementary Figs S7 and S8) [83]. Despite MAGs were sourced from different geographical locations, the rhodopsins are highly conserved (Supplementary Fig. S9) and flanked by highly conserved *Acidiparvus*-like genes (Supplementary Fig. S10, Supplementary Tables S12 and S13). This suggests that the rhodopsins were not acquired through recent HGT events. As the only lineage under this family with streamlined genomes and reduced metabolic capacity, proteorhodopsins may play a role in their survival by augmenting a proton gradient and likely ATP synthesis [82]. A photoheterotrophic lifestyle is commonly reported for other genome streamlined freshwater lineages such as *Nanopelagicales*, *Methylopumilus*, and *Fonsibacter* [3, 6, 10, 65]. However, the latter taxa harbor genes necessary for retinal biosynthesis (*crtY, crtI, crtB, crtE*), while these genes are absent in all *Acidiparvus* MAGs. Despite the lack of retinal chromophore, recent studies showed that rhodopsin can still function through the uptake of retinal from the environment [84] or by a putative unknown retinal biosynthesis pathway [85].

Surprisingly, although having extremely limited metabolic versatility, a complete protoporphyrin-dependent (PPD) heme biosynthesis pathway was found in most of the MAGs in *A. lacustris* and *A. fluvialis* (Figs 4 and 5). A recent study showed that heme auxotrophy is widespread across abundant microbial lineages in aquatic environments, indicating heme reliance in these ecosystems and suggesting heme as public goods in microbial interactions [86, 87]. However, subunits of heme exporters (*ccmABCD*) are only identified in three MAGs, suggesting biosynthesized heme is not exported or may only be released upon phage lysis or natural cell death. Although heme biosynthesis is common to most *Holophagaceae*, *Acidiparvus* has *hemD* (uroporphyrinogen-III synthase) genes that are phylogenetically closer to the genera JADJVL01, JACQZU01 and JAFDVN01, forming a monophyletic cluster, indicating *hemD* is well conserved throughout the lineage (Supplementary Fig. S11). Similar to proteorhodopsins, heme biosynthesis gene clusters are highly conserved (Supplementary Fig. S12) and not flanked by genes from other taxa (Supplementary Tables S14 and S15), indicating that heme biosynthesis is not a result of recent HGT. We hypothesize that heme production among *Acidiparvus* occupies a certain ecological niche in freshwater systems since the whole pathway was kept intact during genome streamlining. Most of the MAGs also encode heme-requiring enzymes such as cbb_3_-type cytochrome *c* oxidase, catalase, peroxidase, and succinate dehydrogenase [86], suggesting these genes give *Acidiparvus* a selective advantage during genome streamlining given this bacterial taxon became auxotrophic for most amino acids and vitamins.

Furthermore, most of the MAGs (57 out of 66) encode either class I cyclopyrimidine dimer photolyase (*phrB*) or catalase-peroxidase (*katG*), both with the ability of using light energy to repair UV-induced DNA damage, suggesting that *Acidiparvus* is adapted to shallow depths in the water column (Supplementary Table S8). This is consistent with metagenomic fragment recruitment and CARD-FISH counts as they are abundant in the epilimnion of lakes while also found in the deeper hypolimnion during mixing. These genes also enable *Acidiparvus* to avoid heme toxicity since most MAGs have complete de novo heme biosynthesis pathway, which can produce reactive oxygen species (ROS) as byproducts [87–89]. Moreover, the detoxification ability of these genes may function as public goods in microbial interactions [86].

Interestingly, 65 out of 66 MAGs harbor cbb_3_-type cytochrome *c* oxidase subunits I-III (*ccoN, ccoO, ccoP)*, forming the minimal functional unit [89] (Supplementary Table S8). These genes were either not identified or incomplete in other *Holophagaceae* lineages (Fig. 5) and any other genome streamlined lineages such as *Nanopelagicales*, *Methylopumilus*, and *Patescibacteria* [5, 10, 90]. We suggest these oxidases have high oxygen affinity, indicating a putative capacity for microaerobic respiration in low O_2_ environments, and play three roles for *Acidiparvus*: firstly, they conserve energy through O_2_ reduction in aerobic respiration. Secondly, *Acidiparvus* might be adapted to microaerobic environments by O_2_ scavenging and, thus, thirdly, expand their distribution throughout the epi- and hypolimnion [28, 91, 92].

Indeed, CARD-FISH shows the presence of *Acidiparvus* down to a depth of 300 m, and metagenomic fragment recruitment indicates that the lineage is abundant at both epi- and hypolimnion (Fig. 1, Supplementary Table S2). Thus, all evidence points toward high flexibility of this lineage to adapt to both surface and deep-water columns.

## Conclusions

We investigated 66 high-quality MAGs of UBA12189, a genus of *Acidobacteriota* with very small genome sizes, distributed in freshwater environments across the world. These MAGs are the only known genome streamlined *Acidobacteriota* so far and the genus appears to be free-living. We propose the new genus name *Acidiparvus* to emphasize its small genome size, with three novel species, whereof two are named *A. lacustris* and *A. fluvialis,* respectively. Common to all MAGs is a reduced metabolic repertoire, resulting in a high degree of auxotrophy for various amino acids, vitamins, and reduced sulfur. When compared to other lineages with reduced genome sizes, *Acidiparvus* has comparatively fewer cell membrane transporters, raising the question of how they can acquire essential nutrients. Despite limited metabolic capacity, *Acidiparvus* encodes proteorhodopsins, a complete heme biosynthesis pathway, heme-requiring enzymes, and cbb_3_-type cytochrome *c* oxidases. These genes are highly conserved, giving *Acidiparvus* a potential selective advantage during the genome streamlining process. We propose them as slow-growing, free-living scavengers in freshwater lakes. Further studies with cultures will be essential as microorganisms are shown to have non-canonical metabolic pathways that cannot be identified with metagenomics studies alone [93].

## Supplementary Material

Supplementary_Table_S1-Metagenomes_ycae124

Supplementary_Table_S2-Recruitment_Results_ycae124

Supplementary_Table_S3-GRiD_ycae124

Supplementary_Table_S4-Single_Copy_Genes_ycae124

Supplementary_Table_S5-MAGs_Statistics_ycae124

Supplementary_Table_S6-All_Reference_MAGs_ycae124

Supplementary_Table_S7-MAGs_BLASTp_HGT_ycae124

Supplementary_Table_S8-Our_MAGs_ORFs_ycae124

Supplementary_Table_S9-CAZy_ycae124

Supplementary_Table_S10-Reference_MAGs_ORFs_ycae124

Supplementary_Table_S11-OPT_Transmembrane_Helices_ycae124

Supplementary_Table_S12-Proteorhodopsin_Neighbouring_Genes_ycae124

Supplementary_Table_S13-Proteorhodopsin_BLASTp_ycae124

Supplementary_Table_S14-hemD_BLASTp_ycae124

Supplementary_Table_S15-hemD_Sequences_ycae124

Supplementary_Table_S16-Pangenome_ycae124

Supplementary_Table_S17-Transporters_ycae124

Supplementary_Table_S18-Unique_Genes_ycae124

Wong_et_al_Supplementary_Information_Accepted_Proof_ycae124

## Data Availability

Fastq files of European freshwater lakes were deposited on European Nucleotide Archive (ENA) under the title: Freshwater metagenomes from different European and Asian lakes (PRJEB35640). The MAGs assembled in the current study were deposited on European Nucleotide Archive (ENA) under accession numbers ERS18353359–ERS18353379. Novel genus and species names were registered at SeqCode [64]. Individual files of Supplementary Table S7 can be found in figshare (https://figshare.com/articles/dataset/BLASTP_results_of_all_contigs/25006274). All other data supporting the findings of this study are available within this paper and its supplementary files.
