# Supplementary material for: Ubiquitous genome streamlined Acidobacteriota in freshwater environments
Source: ISME Commun. 2024 Oct 22;4(1):ycae124. doi: 10.1093/ismeco/ycae124 (PMC11561045; doi:10.1093/ismeco/ycae124)
Supplement: Wong_et_al_Supplementary_Information_Accepted_Proof_ycae124 [file wong_et_al_supplementary_information_accepted_proof_ycae124.docx]

**Supplementary information for Wong et al.**

**Description of the proposed taxa**

Based on our analysis we propose a novel genus under the *Acidobaceriota* family *Holophagaceae*, with two new species. The names have been registered and validated at SeqCode [1].

*Acidiparvus* [A.ci.di.par’vus. N.L. neut. adj. Acidum, sour, acid; N.L. masc. adj. parvus, small, unimportant; N.L. masc. n. Acidiparvus, Acidiparvus, a genus in the acid-loving Acidobacteriota with small genome size.] *Acidiparvus* is the only lineage under *Holophagaceae* to have streamlined genomes and extremely limited metabolic capacities, which resides in aquatic environments. Contains three species, *Acidiparvus* *lacustris*, *Acidiparvus* *fluvialis* and *Acidiparvus* *sp005788175*. The assignment of the novel genus is based on the phylogenetic tree of 46 conserved single copy genes (SCG) of all type genomes of *Holophagaceae* retrieved from Genome Taxonomy Database (GTDB) r207, JGI and a comprehensive metagenomics dataset from freshwater environments [2] (Fig. 1, Supplementary Table S4).

*Acidiparvus* *lacustris* [La.cus’tris, N.L. masc. adj. lacustris, of a lake, resides in freshwater environments, given that most of the genomes of *Acidiparvus* *lacustris* were retrieved from freshwater lakes.]. Represented by ZE-13nov19-LR-8 and sbin449_07_AugE_circ, a long-read genome from Lake Zurich (Switzerland) and a complete genome from Lake Biwa (Japan) respectively [3]. Compared to the other two species, *Acidiparvus* *lacustris* have a higher abundance in the freshwater lakes investigated in the current study.

*Acidiparvus* *fluvialis* [Flu.vi.a’lis, N.L. masc. adj. fluvialis, of a river, describing the bacteria that is exclusively found in rivers.] No MAGs of *Acidiparvus* *fluvialis* were assembled in the current study, and the MAGs with the highest quality is retrieved from Mississippi River (MAG 3300028083-5, Completeness: 94.48%; Contamination: 0.2%). Therefore we propose this assembled genome as the type species.

Although *Acidiparvus* contains three species, we were unable to name the third one due to lack of data. There is so far only one MAG (GCA_005788175) with a genome completeness of 84.86%, which is lower than the required minimum of 90% for submission to The Microbial Genomes Atlas (MiGA) and SeqCode [1, 4]. Therefore, we refer to this species according to the GTDB taxonomy – *Acidiparvus sp005788175*.

**Supplementary text**

**Supplementary Materials and Methods**

**Comparative analysis within the family *Holophagaceae***

Comparative genomic analysis was done between *Acidiparvus* and other *Holophagaceae* lineages. As mentioned in the main text, 237 MAGs including all species representatives of *Holophagaceae* from GTDB r207 [5], a comprehensive metagenomics dataset from freshwater environments [2] and JGI (Joint Genome Institute – Integrated Microbial Genomes) [6] were chosen to construct the phylogenetic tree (Fig. 1). These MAGs were then dereplicated at 95% nucleotide identity using dRep [7], resulting in 41 species-like representative MAGs and culture genomes. Additionally, thirty-three culture genomes of genome-streamlined *Nanopelagicales* [8] and *Methylopumilus* [9] were selected for comparative analysis because they also have small, streamlined genomes, and were isolated from similar environments (i.e. Lake Zurich) in previous sampling campaigns [8, 9]. Thus, this selection of genomes for genomic analyses, i.e., *Acidiparvus* vs. *Holophagaceae* lineages and genome streamlined bacteria sampled from similar environments, provides a comprehensive basis for comparative analyses in both phylogenomics and ecological context.

Proteins in all genomes were predicted using Prodigal v2.6.3 [10]. Subsequently, protein annotations were performed utilizing an in-house pipeline that involved the application of hmmsearch [11] against collections of COG [12], TIGRFAM [13], PFAM [14] hidden markov models (HMMs), and the KOALA algorithm against a non-redundant KEGG database (kofam release 1.3.0) [15]. Gene prediction was discarded if the protein has a minimum coverage of <65% for its entire length to the HMM model, with an e-value threshold of 1e^-3^. Proteins were also annotated using InterProScan [16] with default parameters.

**Pangenome analysis**

The core- and pan-genomes of *Acidoparvus* were computed by comparing all proteins within each genome using BLASTP. An orthologue was defined as having at least 50% identity and 50% coverage.

**Supplementary results and discussion**

**Genome streamlined features of all representative *Acidobacteriota* MAGs**

To determine if *Acidiparvus* is the only genome streamlined lineage under *Acidobacteriota*, we examined all representative species genomes from GTDB r220 [5] classified as *Acidobacteriota* (n = 1891; Supplementary Table S6). Typically, aquatic genome streamlined microbes have common features such as small genome size (<1.8 Mbp), low GC content (< 50%), high coding density (> 90%) and the presence of ion-pumping rhodopsins [17-19]. *Acidiparvus* (g__UBA12189 under current GTDB taxonomy classification) has the smallest genome sizes (Supplementary Table S6). Although genera such as UBA890 and S016-38 have some MAGs with small genome sizes (< 1.8 Mbp), this is not a consistent feature within the respective genera as they also contain genomes with larger genome sizes (i.e. 4.5 Mbp and 2.4 Mbp for UBA890 and S016-38, respectively). All UBA890 and S016-38 do have high coding density (> 90%) but some MAGs have higher GC content (> 60%). Furthermore, none of them encode rhodopsins. Therefore, all evidence points towards *Acidiparvus* being the only aquatic genome-streamlined *Acidobacteriota* genus so far.

**Additional genomic features of *Acidiparvus* and comparative analysis with *Holophagaceae*, *Nanopelagicales* and *Methylopumilus***

All genomes of *Acidiparvus* contain a relatively high fraction of hypothetical proteins comprising 23% - 36% of the genomes (Supplementary Table S5), which is similar to *Nanopelagicales* (22% - 26%) and *Methylopumilus* (19% - 30%). The core-genome of the 66 *Acidiparvus* MAGs encodes only 222 genes (18% - 28% of the assembled genomes), suggesting a higher proportion of flexible genomes with highly diverse auxiliary genes. The pangenome is estimated to contain 1562 genes and the power law regression analysis suggested an almost saturated pangenome for this genus (Supplementary Fig. S6, Supplementary Table S16). Core genes in *Acidiparvus* only comprise 14% of the predicted pangenome, which is significantly lower than that of *Nanopelagicales* and *Methylopumilus* (29% and 48% respectively), suggesting a high strain to strain variability for adaptation during genome streamlining [8, 9].

We further investigated genomic differences of *Acidiparvus* compared to other members of *Holophagaceae* (*Holophaga, Geothrix,* JAFDVN01, JACQZU01, JADJVL01, WRHW01). The main differences of other *Holophagaceae* members are larger genome sizes (2.3 – 4.9 Mbp; Supplementary Table S6), and thus higher metabolic versatility in terms of central carbon metabolisms, nitrogen and sulfur metabolisms, amino acids and vitamins biosynthesis, membrane transporters and motility systems.

Similar to *Acidiparvus*, the other *Holophagaceae* members do not have complete glycolysis and gluconeogenesis pathways except for one *Holophaga* MAG (GCA_019135905). It appears that none of the dereplicated genomes under the family *Holophagaceae* encode phosphoglycerate kinase (*pgk*), which converts D-glycerate 1,3-disphosphate to 3-phosphoglycerate. Furthermore, 11 *Holophagaceae* genomes encode both non-oxidative phase and oxidative phase of pentose phosphate pathway (PPP), providing an alternative to in the incomplete glycolysis pathway (Figure 5).

Most *Geothrix*, *Holophaga*, JAFDVN01, FACQZU01 and JADJVL01 harbor genes involved in assimilatory/dissimilatory nitrate reduction, nitrification, denitrification and assimilatory sulfate reduction at various degrees of completeness (Figure 5, Supplementary Table S16). Three *Holophaga* and two *Geothrix* genomes also encode the complete set of nitrogenase genes (*nifHDK*) and cofactors (*nifNBE*), giving these microbes the ability to fix nitrogen (Supplementary Table S10). None of the aforementioned genes and metabolic pathways were identified in any *Acidiparvus* MAGs. Twenty-three *Holophagaceae* genomes harbor cyanophycinase (*cphB*), allowing them access to additional nitrogen sources.

*Acidiparvus* have significantly limited capacity for amino acid and vitamin biosynthesis. While *Geothrix* and *Holophaga* have the ability to synthesize cysteine, arginine, phenylalanine, tyrosine, tryptophan, serine, threonine, methionine, valine, isoleucine, leucine, lysine, histidine and proline, *Acidiparvus* only have complete pathways for arginine, cysteine and proline synthesis (Figure 5, Supplementary Table S8), rendering it unknown as how this bacterial genus acquires the other amino acids. Regarding vitamin biosynthesis, *Acidiparvus* only have complete pathway for Menaquinone (vitamin K_2_) biosynthesis, while other *Holophagaceae* members are able to produce riboflavin, thiamine, pantothenate and biotin. In summary, *Geothrix* and *Holophaga* have more complete pathways and are prototrophs for many more amino acids and vitamins than *Acidiparvus*.

As described in the main text, *Acidiparvus* have a low number of cell membrane transporters, averaging only 46 transporters per genome (Supplementary Table S17). It appears that other members of the family *Holophagaceae* have higher proportion of transporters, with *Holophaga* and *Geothrix* having 188.3 and 167 transporters per genome respectively. When compared to other freshwater genome streamlined lineages, *Nanopelagicales* [8, 20]***,*** *Nanopelagicus* (acI-B [20]) and *Methylopumilus* [9] have a higher number of transporters (105.4, 118, and 65.2 transporters per genome, respectively) than *Acidiparvus* to compensate for auxotrophies (Supplementary Table S17). However, the relatively high proportions of hypothetical proteins in *Acidiparvus* might also include novel, so far unannotated transporters for nutrient acquisition.

In terms of motility, 32 out of 34 *Geothrix* and *Holophaga* genomes encode the entire flagellar structure, including the basal body (*fliEFGHIJLMNOPQR*), hook (*flgABCDEGHIJ*) and flagellar-hook junction (*flgKL*) (Supplementary Table S10). This grants them motility to potentially avoid predators and search for nutrients. Six genomes contain complete set of genes (*impABCFGHJKLM*, *vasDGJ*, *vgrG* and *hcp*) encoding type VI secretion system (T6SS) (Supplementary Table S10), thus, these microbes are able to deliver effector proteins to other cells, potentially killing competitors and facilitating general secretion to access common goods [21]. None of the aforementioned genes was identified in any of the *Acidiparvus* MAGs, suggesting a free-living and non-motile lifestyle.

As *Acidiparvus* have reduced genomes, we opted to compare the genomic content to other genome streamlined bacteria isolated from similar environments (i.e., *Nanopelagicales* [8] and *Methylopumilus* [9]) to provide a comparison between genome streamlined lineages in similar ecological context. Genes exclusively found in *Acidiparvus* are shown in Supplementary Table S18. Ferredoxin-type protein NapGH (*napGH*) were identified in *Acidiparvus* MAGs, which is essential for electron transfer from ubiquinol to periplasmic nitrate reductase (*napAB*). However, *napAB* were not found in any of the MAGs. Putrescine (a polyamine) production was only identified in *Acidiparvus* but not in *Nanopelagicales* and *Methylopumilus*. It is suggested that amino acid acquisition is different to other genome streamlined bacteria, with *Acidiparvus* obtaining amino acids through amino acid/polyamine antiporters, oligopeptide transporters (OPT) and proton-dependent oligopeptide transporters (TC.POT) rather than ABC transporters such as *liv, opp* and *dpp* amino acid transporters. *Acidiparvus* is the only lineage to encode N-acetylglutamate synthase to convert glutamate to N-acetylglutamate. It is also the only lineage to encode N-acetylornithine carbamoyltransferase (*argF*) and acetylornithine deacetylase (*argE*), allowing them to synthesize arginine from carbamoyl-P. Overall, *Acidiparvus* has a more versatile set of genes for arginine biosynthesis. Long-chain fatty acid transport protein (*fadL*) for transporting hydrophobic compounds across the bacterial membrane was also identified. Only two MAGs have a complete fatty acid degradation pathway, while one-third of the MAGs (23 out of 66) have an almost complete pathway with enoyl-CoA hydratase being absent. For a bacterial symbiont to associate with a host, protein secretion systems are essential [23]. Most of the *Acidiparvus* MAGs contain a partial type II secretion system (T2SS), with most of them encoding components such as secretin (*gspD*), ATPase (*gspE*) and partial inner membrane platform proteins (*gspF*, *gspG*) (Supplementary Table S8). With the absence of outer membrane platform protein (*gspS*), the functionality of T2SS in *Acidiparvus* is uncertain, suggesting this genus was once associated with host(s) but has lost this function through genome streamlining. Although most of the *Acidiparvus* genomes also harbor the *sec* membrane complex (*secD, secF, secG* and *secY*) and *sec*-independent translocase proteins *tatA* and *tatC*, which function to export unfolded proteins through the secretion systems, the complete absence of type III, type IV and type VI secretion systems render the protein secretion pathways non-functional. In other words, there are no evidence that any bacterial secretion systems are present in *Acidiparvus* to interact with any potential host(s), which confirms the CARD-FISH microscopy results that this lineage is free-living and does not attach to any other cells or particles (Fig. 3, Supplementary Fig. S5). This creates a paradox that *Acidiparvus* being metabolically limited, yet free-living independent of any potential hosts and a lower chance to interact with other microorganisms. *Acidiparvus*, however, has the highest proportion of hypothetical, unannotated genes among other genome streamlined lineages analysed in this study. Together with the high number of auxiliary genes in the pangenome, it is suggested that *Acidiparvus* has potentially a high genomic diversity and is thus able to adapt to specialized niches. The extremely reduced metabolic capacity and the high proportion of auxiliary genes creates a fascinating paradox: *Acidiparvus* can do many different things, but they cannot do them alone [23]. *Acidiparvus* is thus potentially a scavenger in aquatic environments, relying on metabolic by-products released by other microbes. This may explain the slow estimated growth rate given the more restricted access to nutrients compared to host- or particle-associated microorganisms.

All genome-streamlined lineages analysed in this study (*Acidiparvus*, *Nanopelagicales* and *Methylopumilus*) encode rhodopsins [8, 9], a common feature of streamlined genomes alongside small genome size, low GC content, high coding density and gene minimalism [19]. As genome streamlined lineages across different phyla retained light-absorbing rhodopsins during the genome streamlining process, it is suggested that rhodopsins helped with the survival of these bacteria upon selection pressure, possibly augmenting energy production through ATP synthesis [24].

**Reference**

1. Hedlund BP, Chuvochina M, Hugenholtz P, Konstantinidis KT, Murray AE, Palmer M, et al. SeqCode: a nomenclatural code for prokaryotes described from sequence data. *Nat Microbiol.* 2022; 7:1702-8.
2. Buck M, Garcia SL, Fernandez L, Martin G, Martinez-Rodriguez GA, Saarenheimo J et al. Comprehensive dataset of shotgun metagenomes from oxygen stratified freshwater lakes and ponds. *Sci Data.* 2021; 8(1):131.
3. Okazaki Y, Nakano S, Toyoda A, Tamaki H. Long-read-resolved, ecosystem-wide exploration of nucleotide and structural microdiversity of lake bacterioplankton genomes. *Microb Ecol.* 2022; 7(4):e00433-22.
4. Rodriguez-R LM, Gunturu S, Harvey WT, Rosselló-Mora R, Tiedje JM, Cole JR, et al. The microbial genomes atlas (MiGA) webserver: taxonomic and gene diversity analysis of Archaea and Bacteria at the whole genome level. *Nucl Acids Res.* 2018; 46(W1):W282-88.
5. Parks DH, Chuvochina M, Rinke C, Mussig AJ, Chaumeil PA, Hugenholtz P. GTDB: an ongoing census of bacterial and archaeal diversity through a phylogenetically consistent, rank normalized and complete genome-based taxonomy. *Nucleic Acids Res.* 2022; 50(D1):D785-94.
6. Chen IM, Chu K, Palaniappan K, Ratner A, Huang J, Huntemann M, et al. THE IMG/M data management and analysis system v. 7: content updates and new features. *Nucl Acid Res*. 2023(D1):D723-D732.
7. Olm MR, Brown CT, Brooks B, Banfield JF. dRep: a tool for fast and accurate genomic comparisons that enables improved genome recovery from metagenomes through de-replication. *ISME J*. 2017; 11(12):2864-68.
8. Neuenschwander SM, Ghai R, Pernthaler J, Salcher MM. Microdiversification in genome-streamlined ubiquitous freshwater Actinobacteria. *ISME J*. 2018; 12(1):185-198.
9. Salcher MM, Schaefle D, Kaspar M, Neuenschwander SM, Ghai R. Evolution in action: habitat transition from sediment to the pelagial leads to genome streamlining in Methylophilaceae. *ISME J*. 2019; 13(11):2764-2777.
10. Hyatt D, Chen GL, LoCascio PF, Land ML, Larimer FW, Hauser LJ. Prodigal: prokaryotic gene recognition and translation initiation site identification. *BMC Bioinformatics*. 2010; 11(1):1-11.
11. Finn RD, Clements J, Eddy SR. HMMER web server: interactive sequence similarity searching. *Nucleic Acids Res.* 2011; 39(suppl_2):W29-37.
12. Tatusov RL, Natale DA, Garkavtsev IV, Tatusova TA, Shankavaram UT, Rao BS, et al. The COG database: new developments in phylogenetic classification of proteins from complete genomes. *Nucleic Acids Res.* 2001; 29(1):22-28.
13. Haft DH, Loftus BJ, Richardson DL, Yang F, Eisen JA, Paulsen IT, et al. TIGRFAMs: a protein family resource for the functional identification of proteins. *Nucleic Acids Res.* 2001; 29(1):41-43.
14. Mistry J, Bateman A, Finn RD. Predicting active site residue annotations in the Pfam database. *BMC Bioinformatics*. 2007; 8(1):1-4.
15. Kanehisa M, Sato Y, Kawashima M, Furumichi M, Tanabe M. KEGG as a reference resource for gene and protein annotation. *Nucleic Acids Res.* 2016; 44(D1):D457-D462.
16. Jones P, Binns D, Chang HY, Fraser M, Li W, McAnulla C, et al. InterProScan 5: genome-scale protein function classification. *Bioinformatics*. 2014; 30(9):1236-1240.
17. Giovannoni SJ, Thrash CJ, Temperton B. Implications of streamlining theory for microbial ecology. *ISME J*. 2014; 8(8): 1553-65.
18. Cho BCM Hardies SC, Jang GI, Hwang CY. Complete genome of streamlined marine actinobacterium Pontimonas salivibrio strain CL-TW6 T adapted to coastal planktonic lifestyle. *BMC Genomics*. 2018; 19: 1-21.
19. Chiriac MC, Haber M, Salcher MM. Adaptive genetic traits in pelagic freshwater microbes. *Environ Microbiol.* 2023; 25(3):606-641.
20. Garcia SL, McMahon KD, Martinez-Garcia M, Srivastava A, Sczyrba A, Stepanauskas R, et al. Metabolic potential of a single cell belonging to one of the most abundant lineages in freshwater bacterioplankton. *ISME J*. 2013; 7(1):137-147.
21. Allsopp LP, Bernal P. Killing in the name of: T6SS structure and effector diversity. *Microbiology*. 2023; 169(7):001367.
22. Chiriac MC, Bulzu PA, Andrei AS, Okazaki Y, Nakano SI, Haber M, et al. Ecogenomics sheds light on diverse lifestyle strategies in freshwater CPR. *Microbiome.* 2022; 10(1):1-21.
23. Garcia SL, Buck M, Hamilton JJ, Wurzbacher C, Grossart HP, McMahon KD, et al. Model communities hint at promiscuous metabolic linkages between ubiquitous free-living freshwater bacteria. *MSphere.* 2018; 3(3):e00202-18.
24. Béja O, Aravind L, Koonin EV, Suzuki MT, Hadd A, Nguyen LP, et al. Bacterial rhodopsin: evidence for a new type of phototrophy in the sea. *Science*. 2000; 289(5486):1902-6.

**Supplementary Figures and Tables**

**
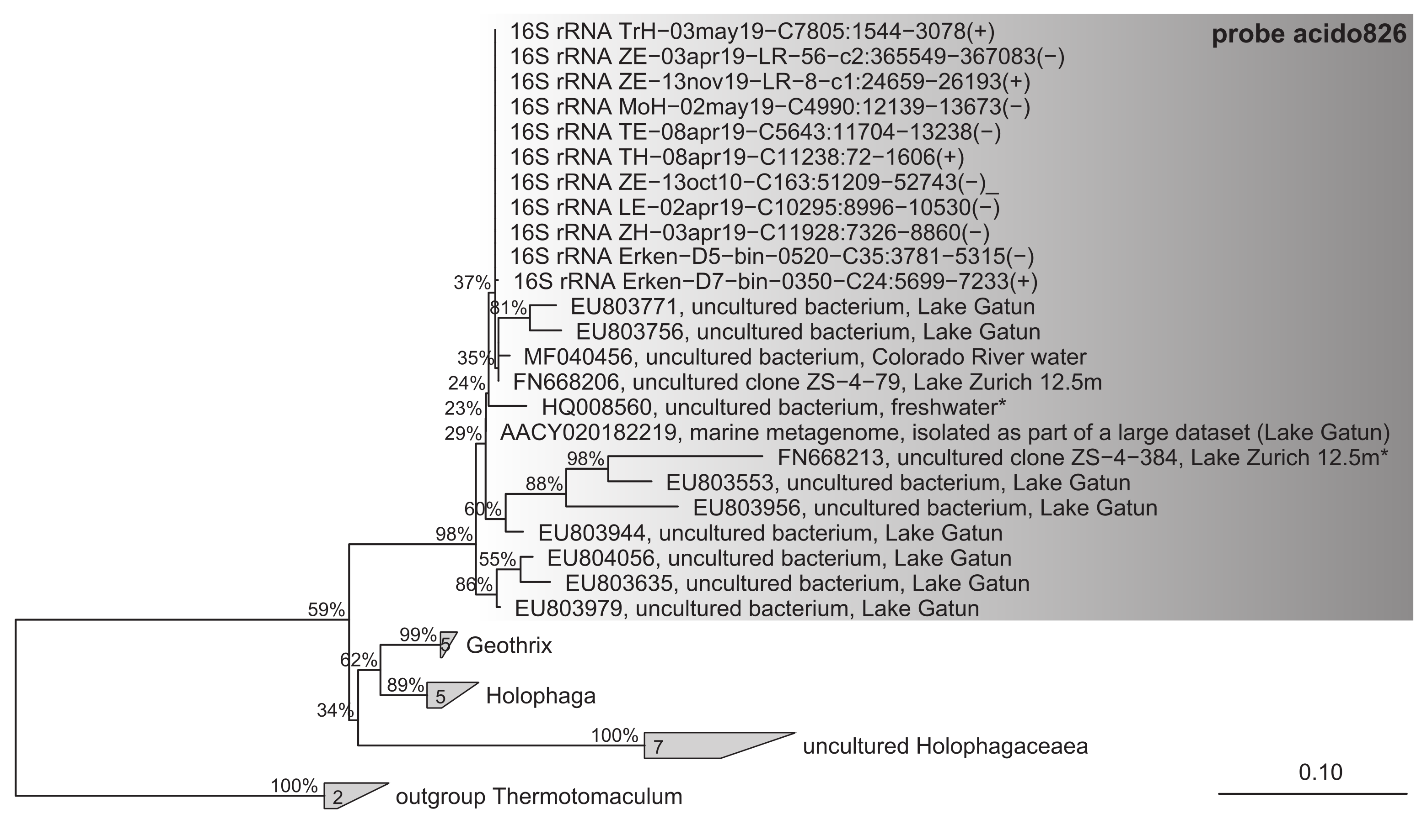
**

**Supplementary Fig. S1.** CARD-FISH probe tree. Randomized Axelerated Maximum Likelihood tree (RAxML, 100 bootstraps, GTR-GAMMA model) of 16S rRNA gene sequences affiliated with *Holophagaceae*. Probe acido826 (highlighed in grey) targets all sequences affiliated to *Acidiparvus* (marine group Holophagales_Holophagaceae in SILVA classification) and was used for visualizing *Acidiparvus* cells by CARD-FISH.


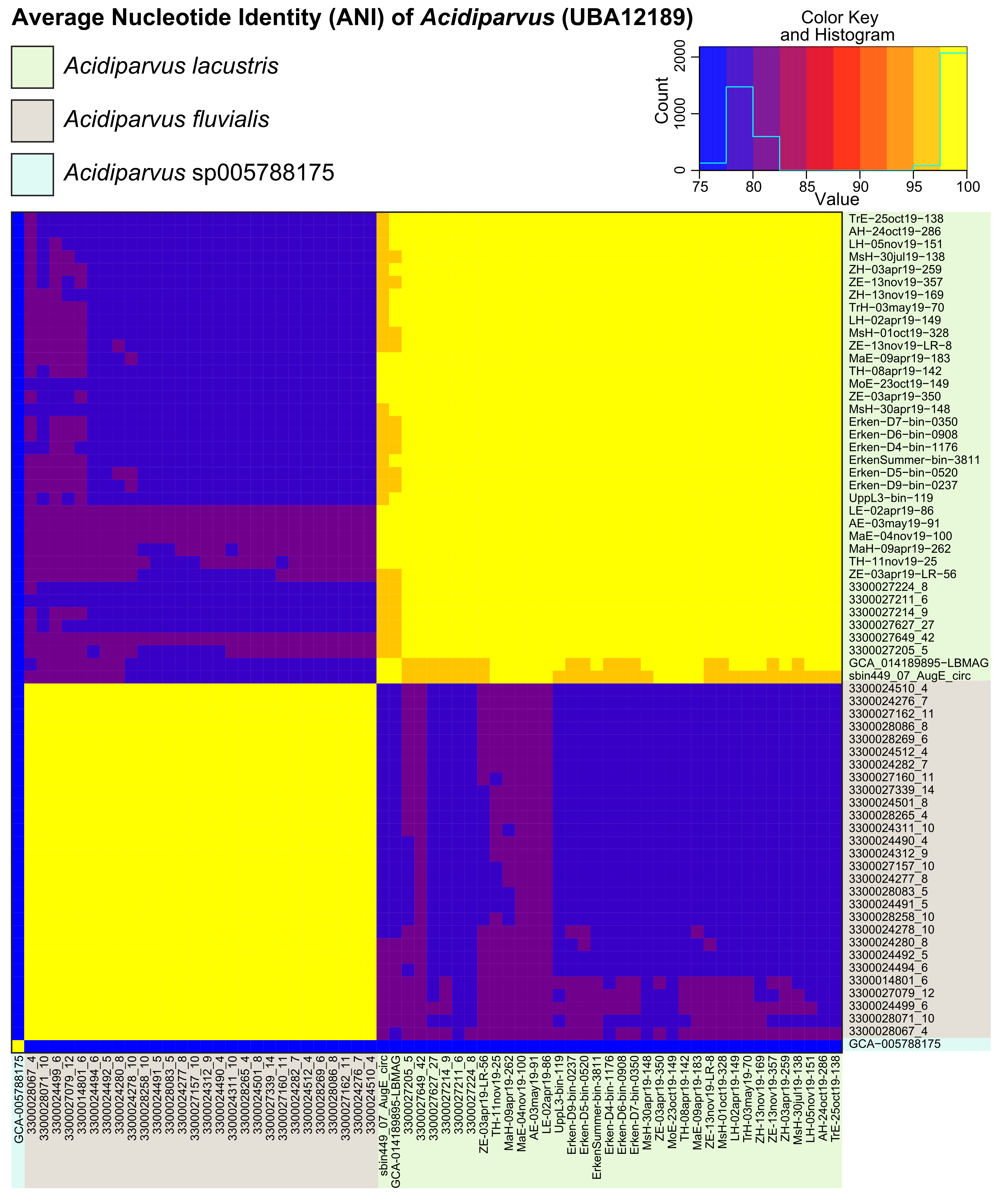


**Supplementary Fig. S2.** Comparison of the average nucleotide identity (ANI) across all 66 MAGs of the genus UBA12189, showing 3 distinct phylogenetic clades of different species (ANI < 95%).

**Supplementary Fig. S3.** Comparison of the average amino acid identity (AAI) across all 66 MAGs of the genus UBA12189, showing 3 distinct phylogenetic clades of the same genus (AAI > 65%).


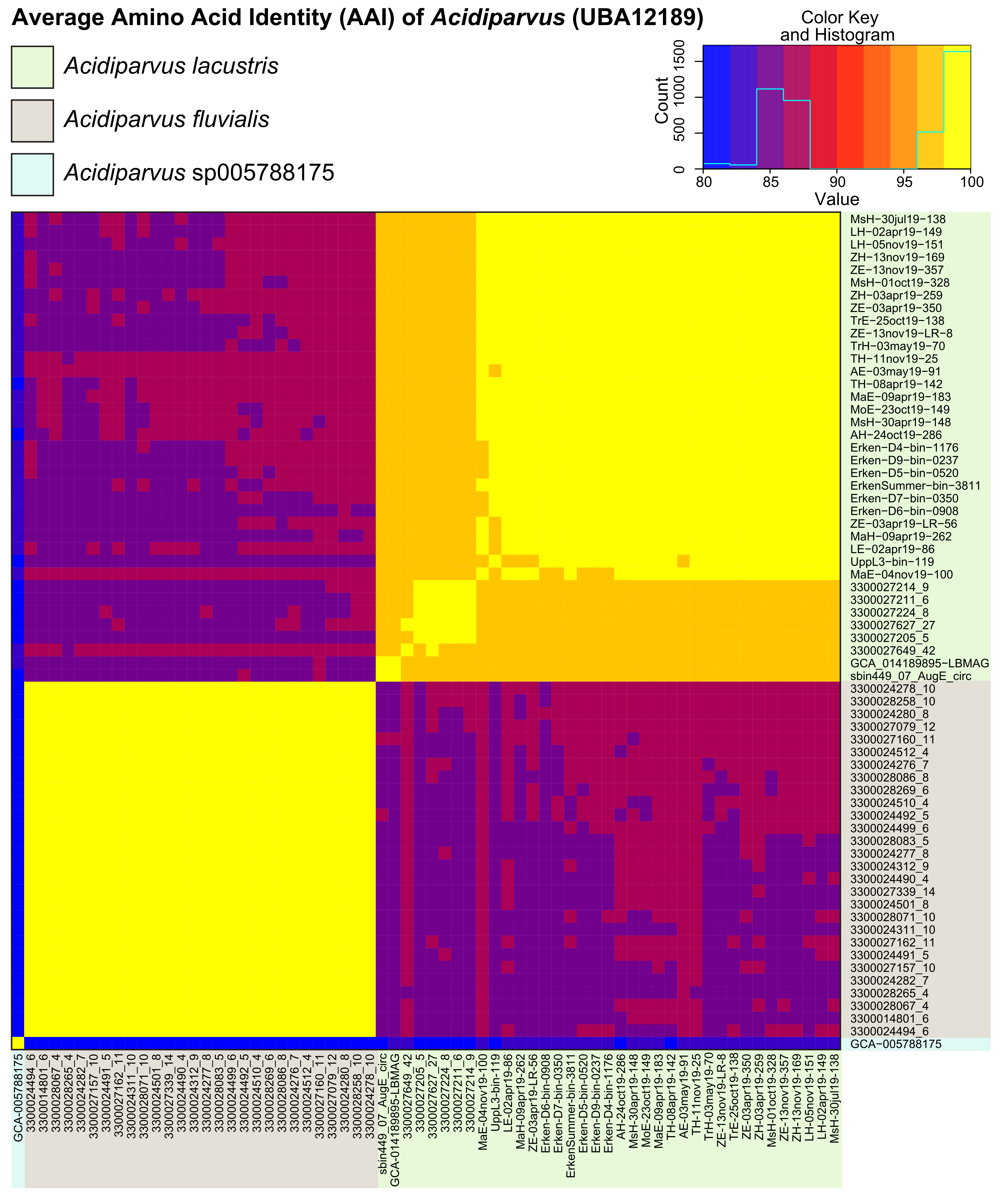


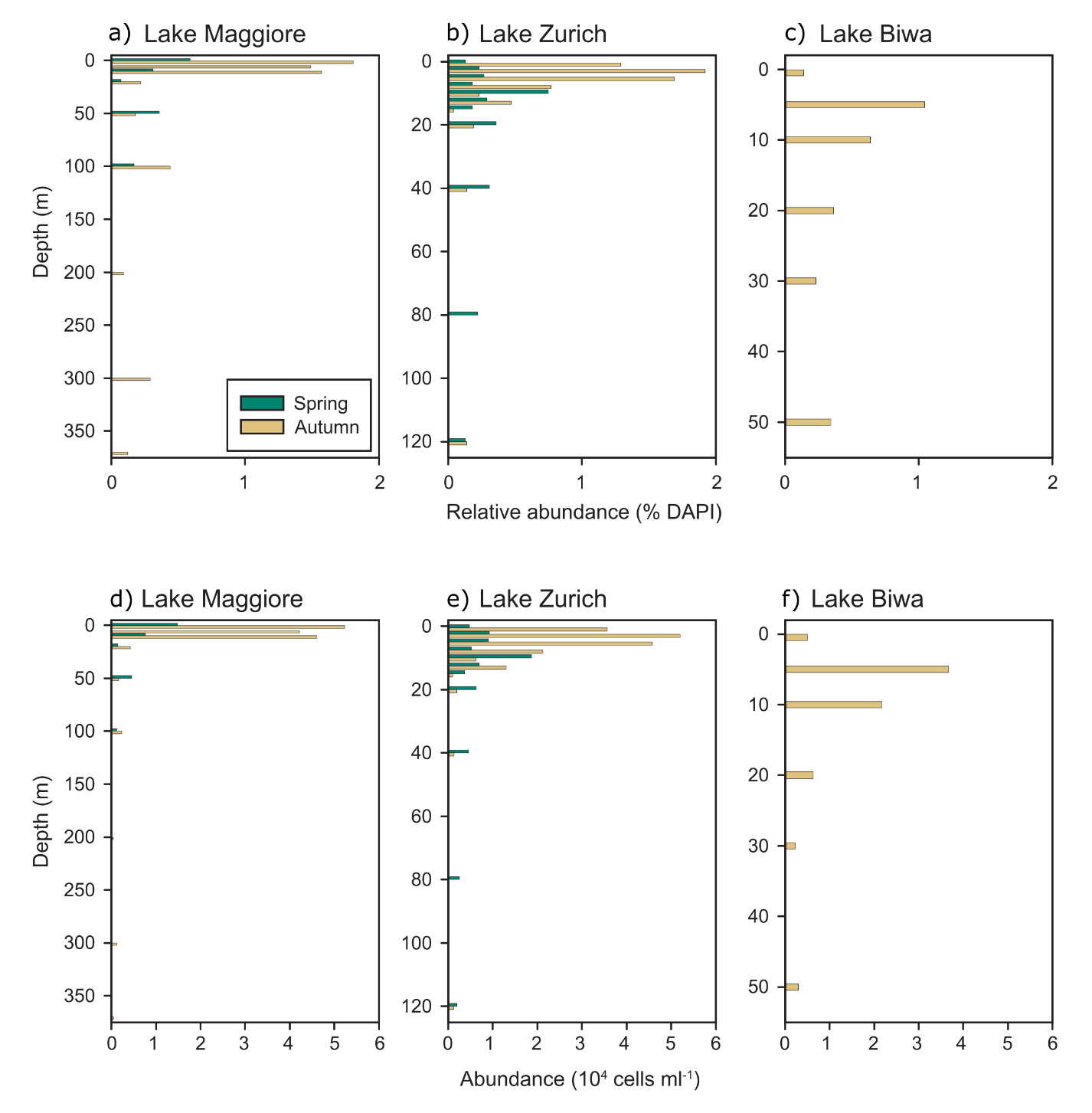


**Supplementary Fig. S4.** Abundance of *Acidiparvus* in different seasons and water depths determined by CARD-FISH and flow cytometry. **a-c.** Relative abundances (% of all DAPI stained prokaryotes) of *Acidiparvus* in different depths of Lakes Maggiore, Zurich and Biwa during spring and autumn, respectively. **d-f.** Total average abundances (10^4^ cells ml^-1^) of UBA12189 in different depths of Lake Maggiore, Zurich and Biwa during spring and autumn, respectively. Abundances were calculated by multiplying relative abundances with total prokaryotic counts determined by flow cytometry. In Lake Biwa only samples from autumn are available.

**
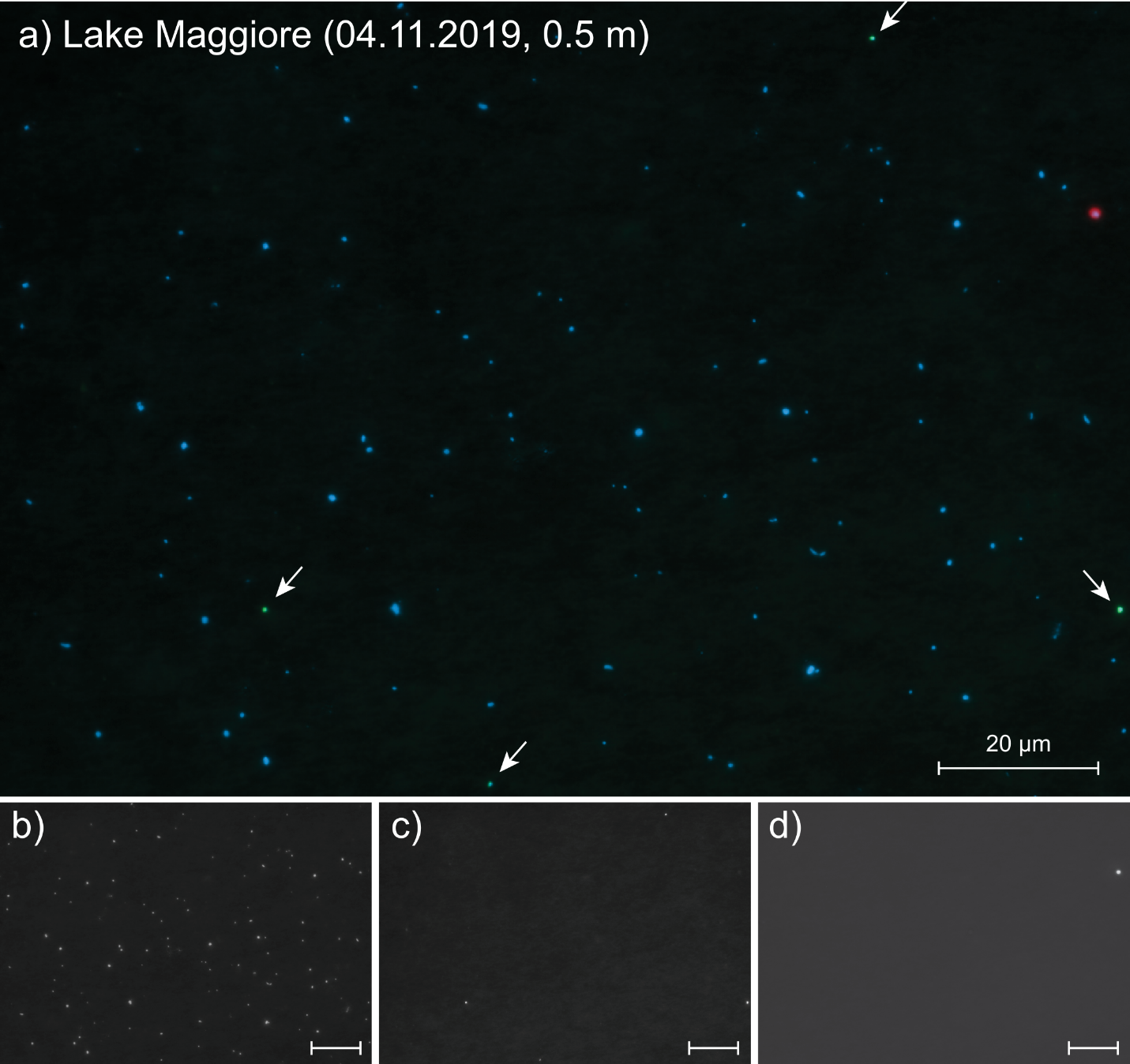
**

**Supplementary Fig. S5.** CARD-FISH imaging of *Acidiparvus* in Lake Maggiore. **a.** Overlay image of CARD-FISH (green), DAPI (blue) and autofluorescence (red) signals from a sample from Lake Maggiore (0.5 m depth, November 4, 2019). **b-d.** Individual images of DAPI stained prokaryotes **(b)**, CARD-FISH stained *Acidiparvus* **(c)** and autofluorescence **(d)** from the same field of view. *Acidiparvus* cells are highlighted by arrows, the scale bar in all images represents 20 µm.


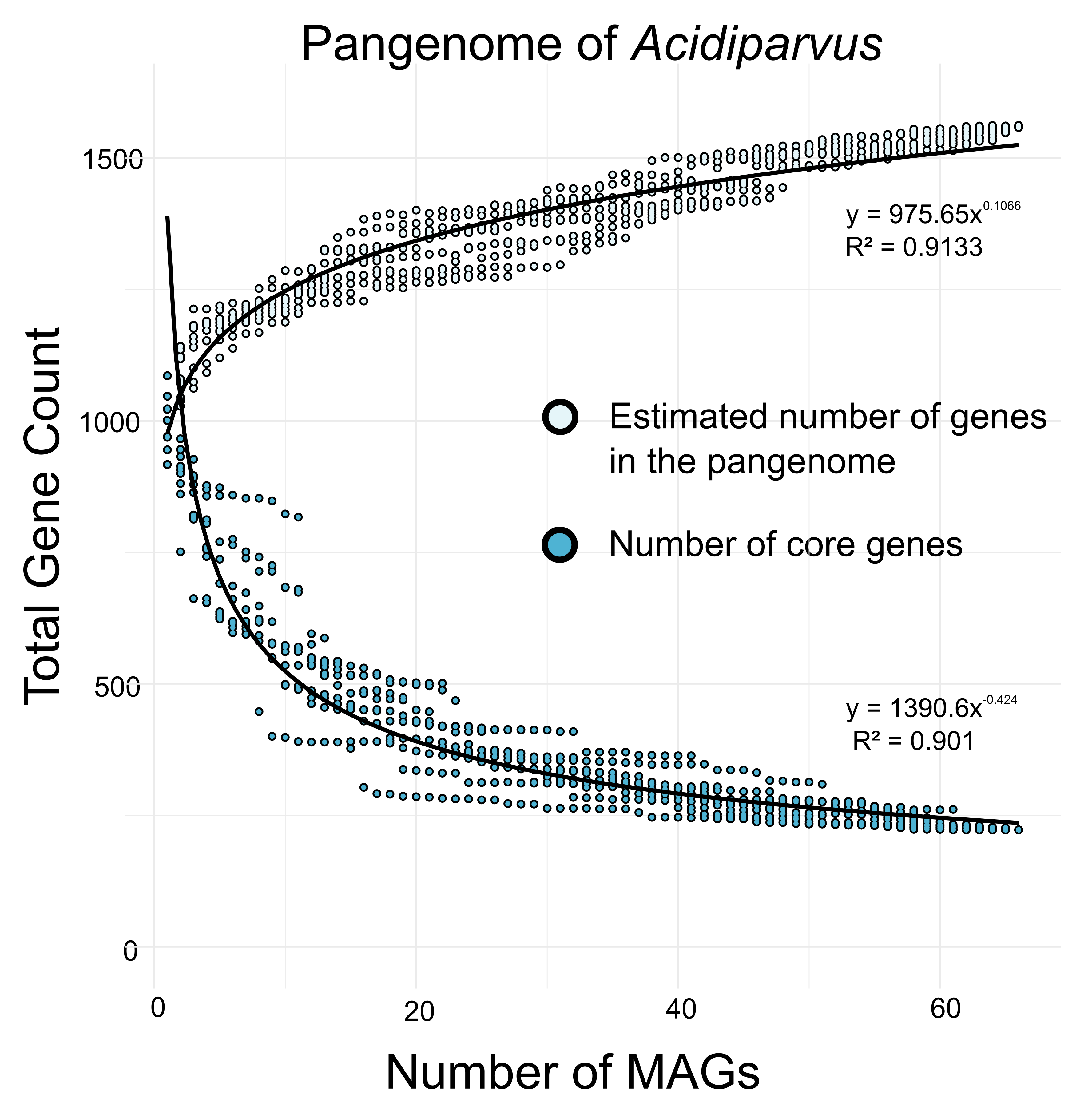


**Supplementary Fig. S6.** Pangenome and core-genome analysis of *Acidiparvus*. Regression lines were fitted with two-parameter power law regression (for pangenome) and exponential decay (for core-genome). Estimated total gene count and predicted core gene count are listed in Supplementary Table S16.

**
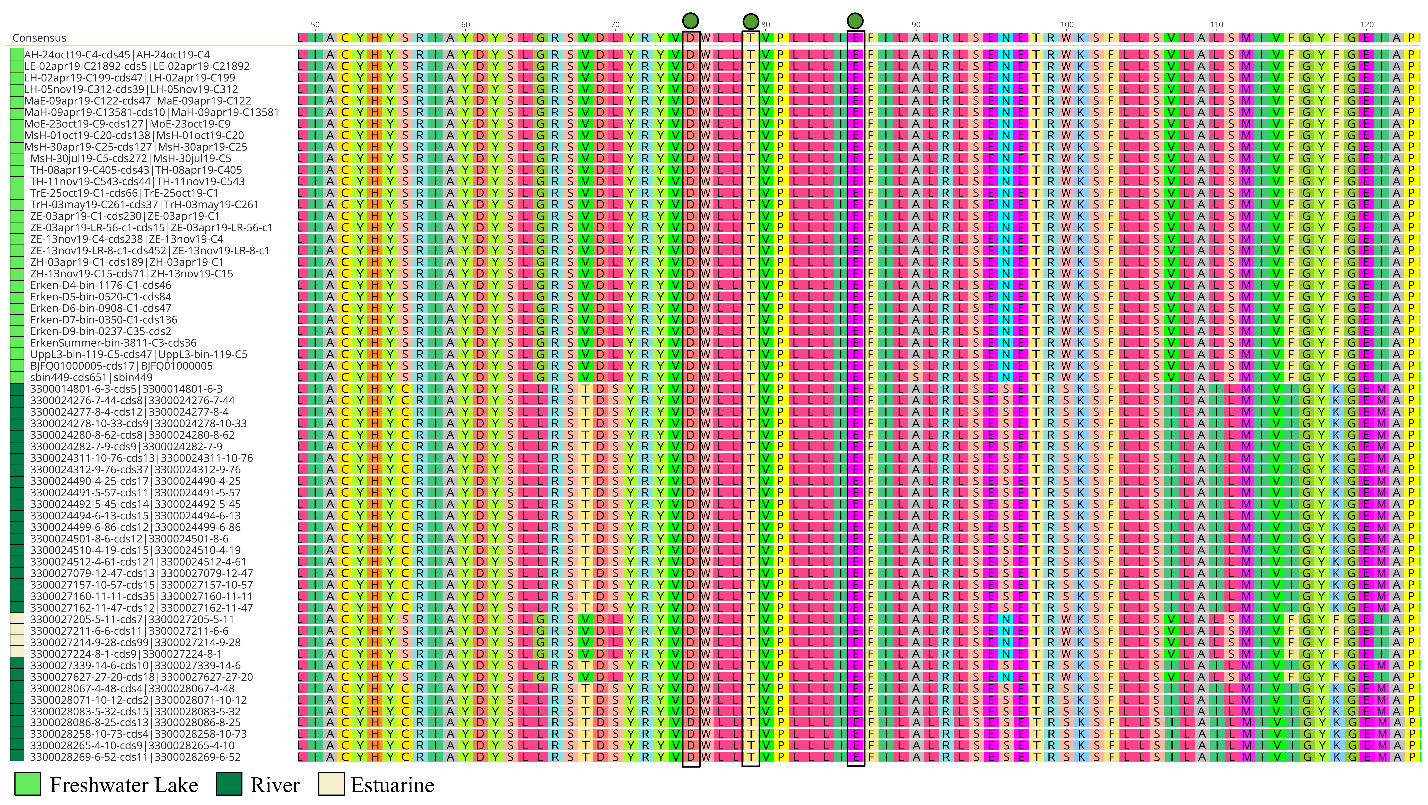
**

**Supplementary Fig. S7.** Helix C of proteorhodopsins found in *Acidiparvus* MAGs. The green circles indicate the DTE motif, which is the proton pumping motif of proteorhodopsins.

**
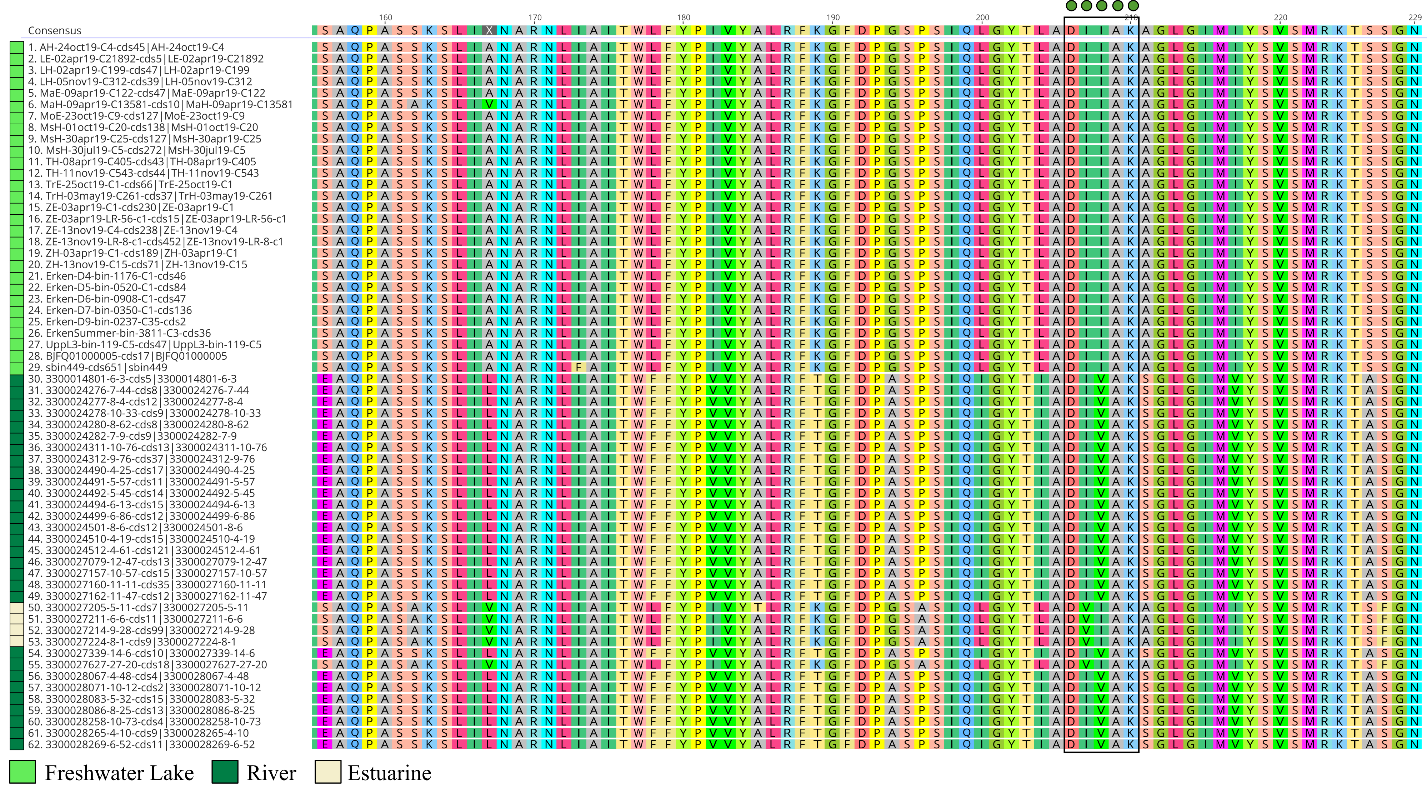
**

**Supplementary Fig. S8.** Helix G of proteorhodopsins found in *Acidiparvus* MAGs. The green circles indicate the DxxxK motif, which is responsible for light sensitivity.

**
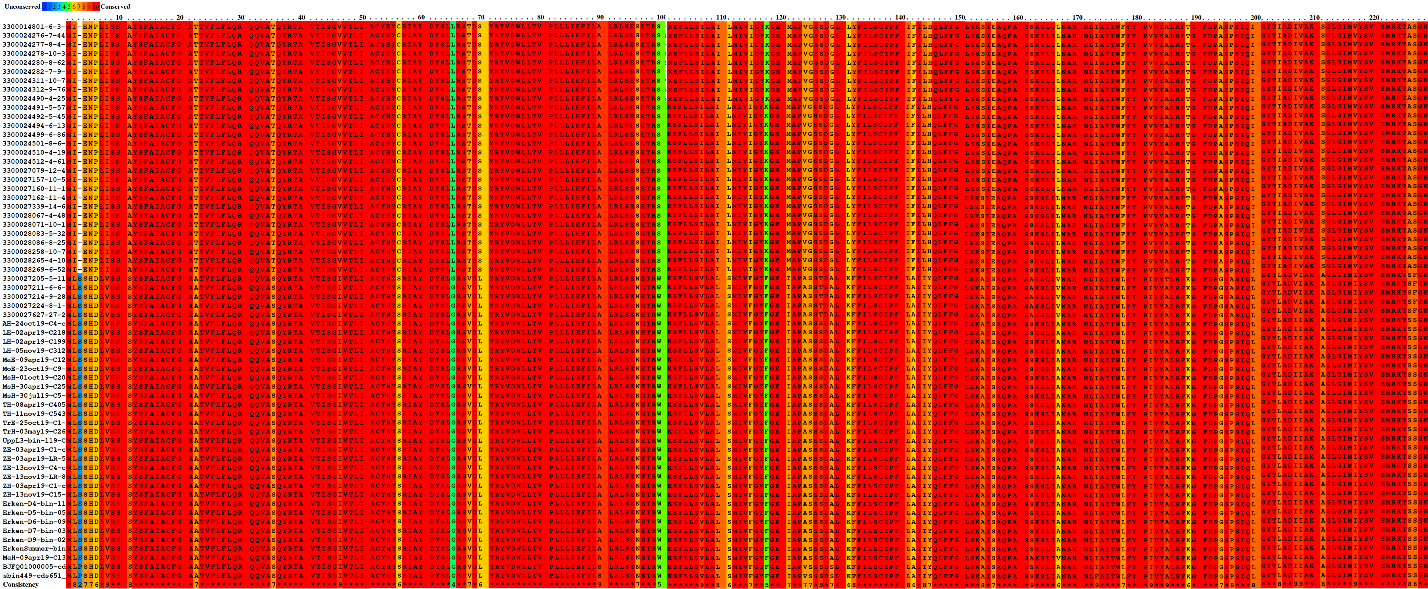
**

**Supplementary Fig. S9.** Praline results showing conserved sequences of *Acidiparvus* proteorhodopsins.

**
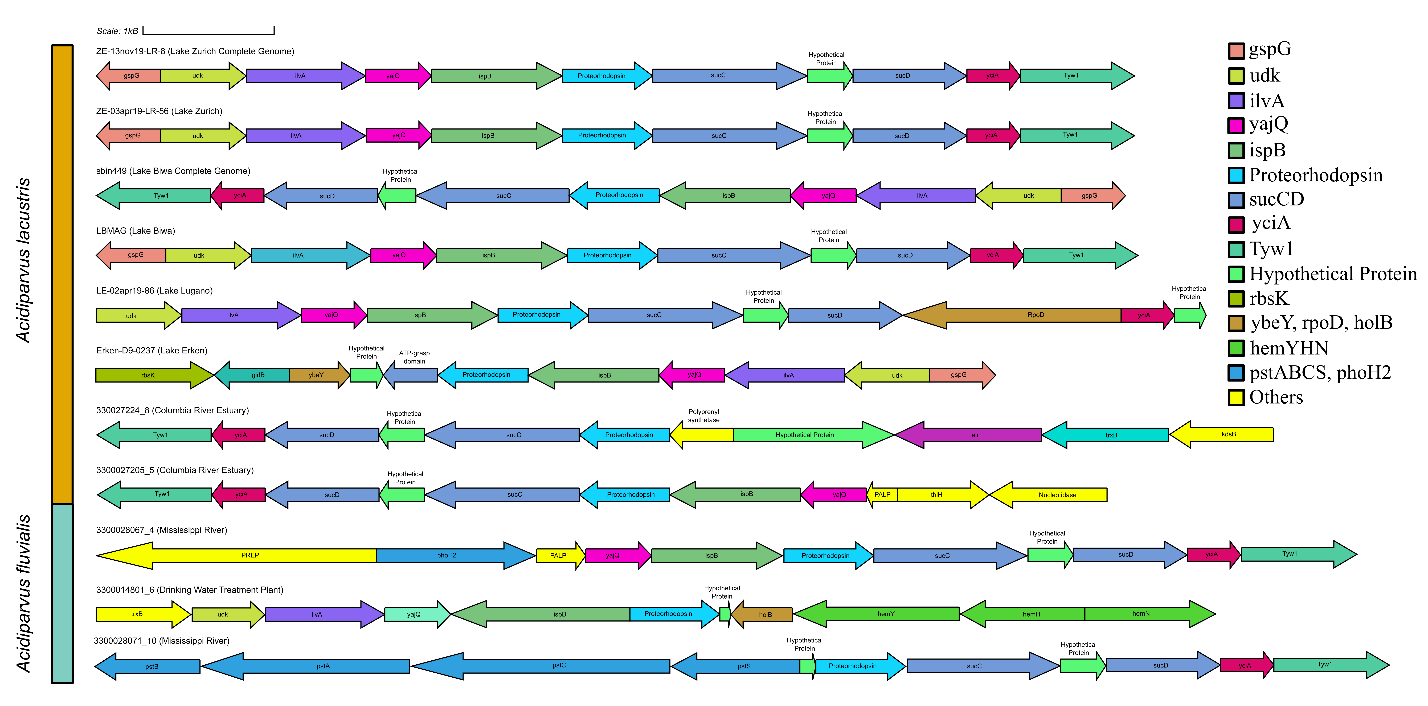
**

**Supplementary Fig. S10**. Proteorhodopsin and the neighbouring genes in *Acidiparvus* genomes. Abbreviations: *gspG*—general secretion pathway protein G, *udk*—uridine kinase, *ilvA*—threonine dehydratase, *yajQ*—cyclic-di-GMP-binding protein, *ispB*—octaprenyl-diphosphate synthase, *sucC*—succinyl-CoA synthetase beta subunit, *sucD*—succinyl-CoA synthetase alpha subunit, *yciA*—acyl-CoA thioesterase YciA, *tyw1*—tRNA 4-demethylwyosine synthase, *rbsK*—ribokinase, *ybeY*—probable rRNA maturation factor, *rpoD*—RNA polymerase primary sigma factor, *holB*—DNA polymerase III subunit delta, *hemY*—coproporphyrinogen III oxidase, *hemN*—oxygen-independent coproporphyrinogen III oxidase, *hemH*—coproporphyrin ferrochelatase, *pstABCS*—phosphate transport system, *phoH2*—PhoH-like ATPase, *kdsB*—3-deoxy-manno-octulosonate cytidylyltransferase, *PREP*—prolyl oligopeptidase, *PALP*—Pyridoxal-phosphate dependent enzyme, *trxB*—thioredoxin reductase (NADPH), *thiH*—2-iminoacetate synthase.

**
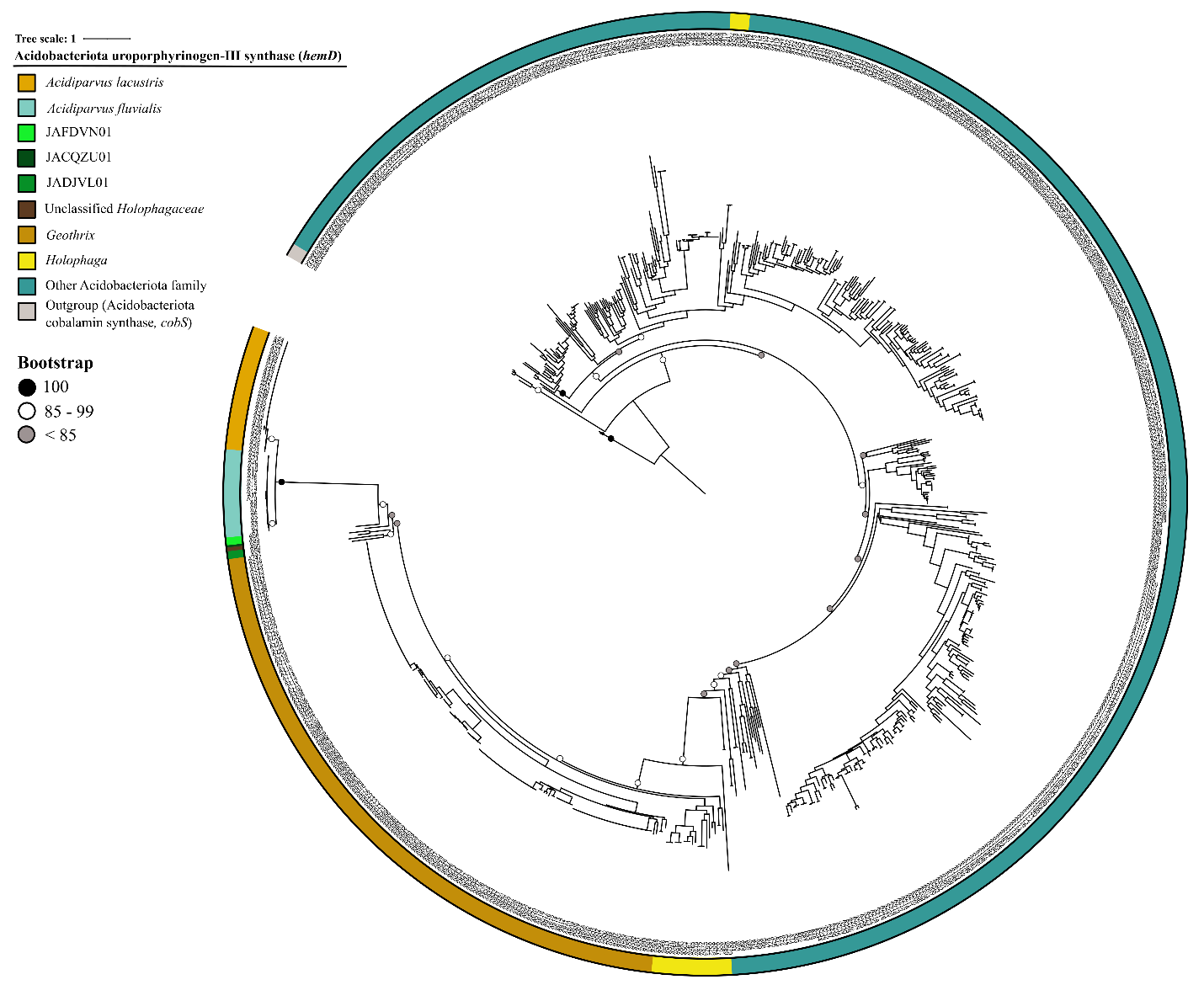
**

**Supplementary Fig. S11.** Maximum likelihood phylogenetic tree with LG+F+G4 model of all *Acidobacteriota* *hemD* genes retrieved from NCBI. The tree shows that *hemD* from *Acidiparvus* are monophyletic and are phylogenetically closer to those from JAFDVN01, JACQZU01, JADJVL01 than *Geothrix* and *Holophaga*. Color panel represents taxonomy of *Acidobacteriota* in which *hemD* genes were extracted. Labels are listed in Supplementary Table S15.

**
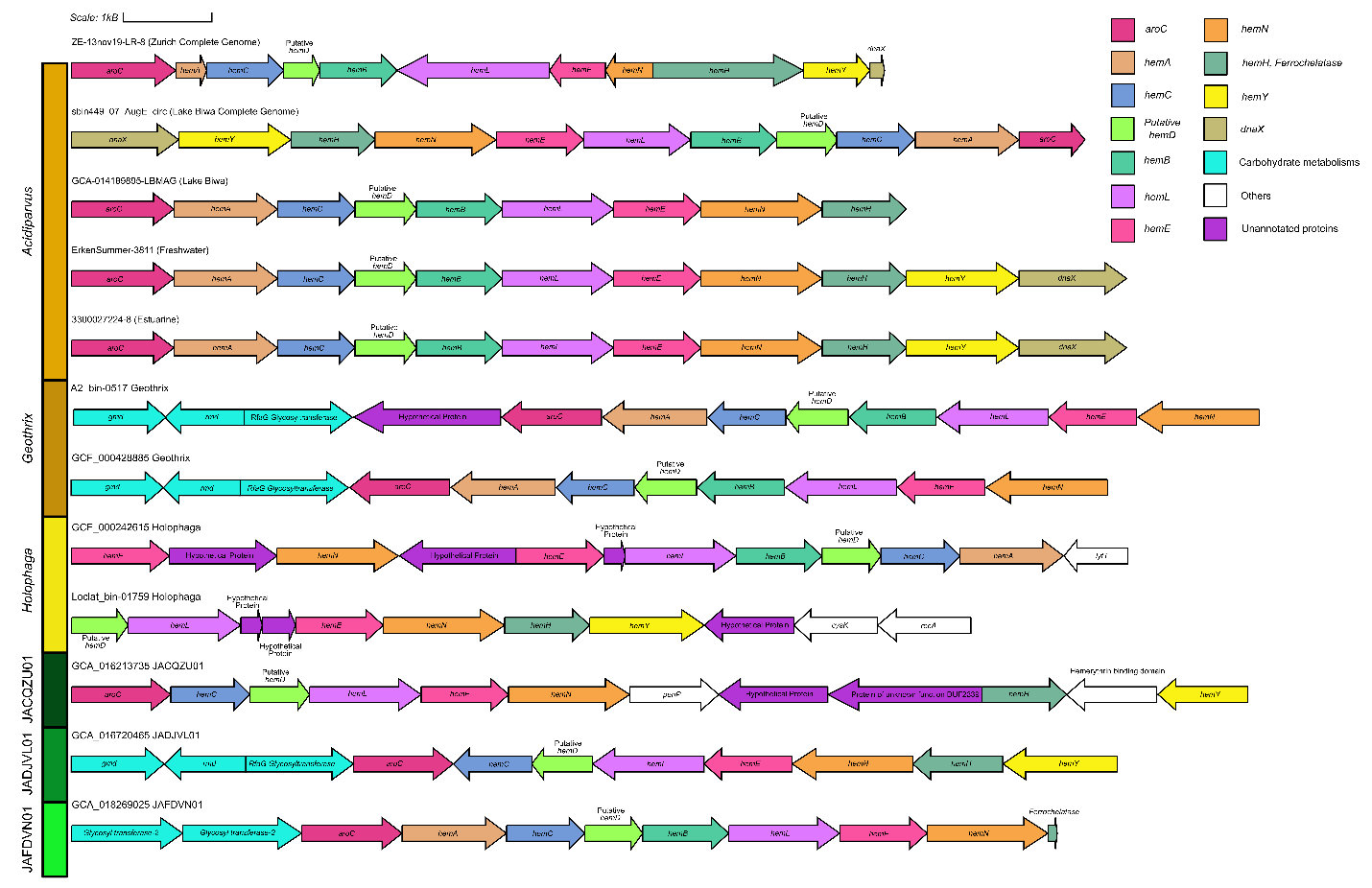
**

**Supplementary Fig. S12.** Uroporphyrinogen-III synthase (*hemD*) and the surrounding genes in *Acidiparvus* genomes. Color panel on the left represents lineages of *Holophagaceae.* Abbreviations: *aroC*—chorismate synthase, *hemA*—glutamyl-tRNA reductase, *hemC*—hydroxymethylbilane synthase, *hemB*—porphobilinogen synthase, *hemE*—uroporphyrinogen decarboxylase, *hemL*—glutamate-1-semialdehyde 2,1-aminomutase, *hemN*—oxygen-independent coproporphyrinogen III oxidase, *hemH*—coproporphyrin ferrochelatase, *hemY*—coproporphyrinogen III oxidase, *dnaX*—DNA polymerase III subunit gamma/tau, *rmd*—GDP-4-dehydro-6-deoxy-D-mannose reductase, *gmd*—GDP-D-mannose dehydratase, *lytT*—two-component system, LytTR family, response regulator, *cysK*—cysteine synthase, *recA*—recombination protein RecA, *penP*—beta-lactamase class A.

**Supplementary Table S1.** Details of the metagenomes analysed in this study.

**Supplementary Table S2.** Recruitment results (coverage per Gbp) for all MAGs versus all samples analyzed in this study. Only metagenomes that have an average of > 0.1 coverage per Gbp were included.

**Supplementary Table S3.** Estimated replication rate using GRiD. Only MAGs obtained from our sampling trips and Lake Biwa were analysed.

**Supplementary Table S4.** List of 46 single copy genes (SCGs; the minimum number of SCGs in every MAGs) selected in the MAGs to construct the phylogenetic tree. The table also includes the labels for Fig. 1.

**Supplementary Table S5.** General statistics of MAGs analysed in this study.

**Supplementary Table S6.** Genome statistics of all species representative genomes of *Acidiobacteriota*.

**Supplementary Table S7.** Percentage of *Acidiparvus* genes in each MAG determined by Blastp.

**Supplementary Table S8.** Summary of open reading frames (ORF) in all *Acidiparvus* genomes with gene annotation against KEGG, COG, PFAM and TIGRFAM.

**Supplementary Table S9.** Abundance of carbohydrate-active enzymes (CAZy) in each *Acidiparvus* MAG.

**Supplementary Table S10.** Summary of open reading frames (ORF) in all dereplicated *Holophagaceae, Nanopelagicales* and *Methylopumilus* genomes with gene annotation against KEGG, COG, PFAM and TIGRFAM.

**Supplementary Table S11.** Number of transmembrane helices in hypothetical proteins and oligopeptide transporter proteins (OPT) in *Acidiparvus*.

**Supplementary Table S12.** Summary of neighbouring genes next to proteorhodopsin in *Acidiparvus*.

**Supplementary Table S13.** Blastp results of proteorhodopsin and neighbouring genes. Results shows that the rhodopsin contigs are highly conserved and flanked by highly conserved *Acidiparvus*-like genes.

**Supplementary Table S14.** Blastp results of hemD and neighbouring genes. Results shows that the hemD contigs are highly conserved and flanked by highly conserved *Acidiparvus*-like genes.

**Supplementary Table S15.** Labels for Supplementary Figure S11 and the *Acidiparvus* hemD sequences used to construct the phylegenetic tree in Supplementary Figure S11

**Supplementary Table S16.** Pangenome analysis of UBA12189, with estimated total gene count in the pangenome and predicted core genes.

**Supplementary Table S17.** Number and percentage of membrane transporter systems in *Acidiparvus, Holophaga, Geothrix* and other genome-streamlined bacteria (*Nanopelagicales, Methylopumilus*, acI-B1).

**Supplementary Table S18.** Genes found exclusively in *Acidiparvus* when compared to other aquatic genome-streamlined bacteria such as *Nanopegicales* and *Methylopumilus*.

**List of abbreviations used in the main text:**

**AAI:** Average amino acid identity

**ANI:** Average nucleotide identity

**CARD-FISH:** Fluorescence *in situ* hybridization followed by catalyzed reporter deposition

**CAZy:** Carbohydrate-active enzymes

**DAPI:** 4‘6-diamidino-2-phenylindole

**ETC:** Electron transport chain

**G3P:** Glyceraldehyde 3-phosphoate

**GC content:** Guanine-Cytosine content

**HGT:** Horizontal gene transfer

**MAG:** Metagenome-assembled genomes

**MFS:** Major facilitator superfamily transporter

**OPT:** Oligopeptide transporters

**PPP:** Pentose phosphate pathway

**SCG:** Single copy genes

**T2SS:** Type II secretion system

**TCA:** Tricarboxylic acid

**TC.APA:** Basic amino acid/polyamine antiporters

**TC.POT:** Proton-dependent oligopeptide transporters

**ROS:** Reactive oxygen species

* Abbreviations used in figures are explained in figure legends
